# Two-Photon Absorption: An Open Door to the NIR-II Biological Window?

**DOI:** 10.3389/fchem.2022.921354

**Published:** 2022-06-24

**Authors:** Paige A. Shaw, Ewan Forsyth, Fizza Haseeb, Shufan Yang, Mark Bradley, Maxime Klausen

**Affiliations:** EaStCHEM School of Chemistry, University of Edinburgh, Edinburgh, United Kingdom

**Keywords:** two-photon absorption, infrared dyes, fluorescent imaging, near-infrared II, two-photon microscopy, tissue penetration, pulsed lasers

## Abstract

The way in which photons travel through biological tissues and subsequently become scattered or absorbed is a key limitation for traditional optical medical imaging techniques using visible light. In contrast, near-infrared wavelengths, in particular those above 1000 nm, penetrate deeper in tissues and undergo less scattering and cause less photo-damage, which describes the so-called “second biological transparency window”. Unfortunately, current dyes and imaging probes have severely limited absorption profiles at such long wavelengths, and molecular engineering of novel NIR-II dyes can be a tedious and unpredictable process, which limits access to this optical window and impedes further developments. Two-photon (2P) absorption not only provides convenient access to this window by doubling the absorption wavelength of dyes, but also increases the possible resolution. This review aims to provide an update on the available 2P instrumentation and 2P luminescent materials available for optical imaging in the NIR-II window.

## 1 Introduction

**FIGURE 1:**
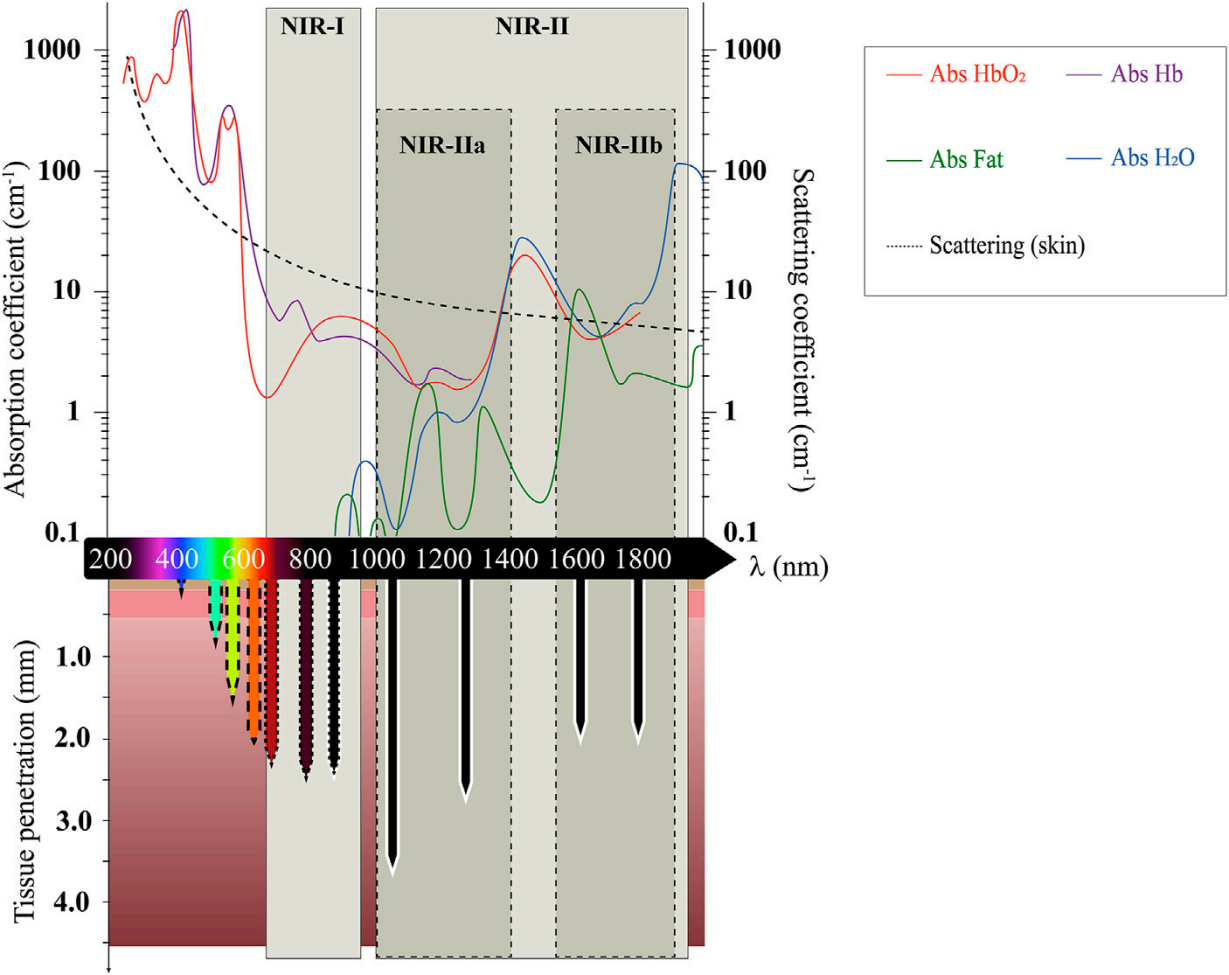
Absorption and scattering coefficients of endogenous chromophores, tissues, and water over the visible and SWIR wavelengths (200–2000 nm). The downwards arrows represent the tissue penetration of light at these wavelengths, according to the values reported for human skin in ref. (Bashkatov et al., 2005). Dashed, dotted and solid outlines on penetration arrows represent the increase in imaging resolution with increased wavelengths. Adapted from values reported in ref (Jacques, 2015). and references therein.

Optical molecular imaging (OMI) technologies such as fluorescence imaging, Raman imaging, and optical coherence tomography, have emerged as safe and non-invasive tools to screen and monitor and diagnose disease in real-time, and follow treatment progress (Nicolson et al., 2021; Cao et al., 2019). Fluorescence-based OMI offers the ability to investigate biological systems with high spatio-temporal resolution and is now commonly applied to allow bio-molecular detection, drug distribution monitoring, image-guided surgery, and clinical diagnosis and therapy (Diao et al., 2015). The majority of *in vivo* fluorescence imaging approaches are performed using visible (400 nm–700 nm) and near-infrared I (NIR-I, 700 nm–900 nm) light due to the availability of light sources and detectors operating in this regime. However, the optical properties of tissues in this range of wavelengths intrinsically generate two challenges: a loss of signal due to poor penetration of light, and a low signal to background ratio (SBR) resulting from tissue auto-fluorescence (Zhang et al., 2016). The poor penetration of light *in vivo* arises due to the strong attenuation coefficients of tissue components, which causes photons to be scattered or absorbed by endogenous chromophores as they travel through tissues (Figure 1) (Keiser and Keiser, 2016). Tissue auto-fluorescence also represents a major limitation when imaging at shorter wavelengths. This loss in signal along with low SBR both contribute to the reduction in the resolution of the output image with increased depth, thereby limiting the optical imaging to micrometre depths.

Favorably, the spectral properties of biological tissues are strongly wavelength-dependent, which opens two windows of “biological optical transparency” that enable higher resolution fluorescence imaging (Nicolson et al., 2021; Cao et al., 2019; Sordillo et al., 2014a; Kenry et al., 2018; Chen et al., 2021; Hassan and Klaunberg, 2004; Li and Wang, 2018). Firstly, the absorption coefficients of tissue components such as whole blood (oxygenated HbO_2_ or deoxygenated Hb), or fat (Figure 1) strongly decrease when reaching red/NIR wavelengths, which constitutes a first “NIR-I″ transparency window (Bashkatov et al., 2005). Secondly, and more significantly, as the scattering coefficient of light is inversely proportional to the fourth power of its wavelength, photons of even longer wavelengths are more likely to continue on their intended linear trajectory rather than being scattered away due to their interactions within the tissue (Sordillo et al., 2014a; Wang et al., 2020a; Lockwood and Luo, 2016). For this reason, the use of low-energy light in the so-called “near-infrared II” (NIR-II) window (1000 nm–2000 nm), also known as the short-wave infrared region (SWIR), can not only help achieve higher penetration in biological tissue (Figure 1), but also aid in enhanced spatio-temporal resolution at fixed tissue depths, as well as reduce risks of photo-toxicity (Wang et al., 2020a; Ding et al., 2018). Moreover, tissue auto-fluorescence generated by endogenous molecules such as flavins, NADH, porphyrins, and collagens decreases significantly at longer wavelengths where these fluorophores do not absorb. Substantially diminished background auto-fluorescence is observed with NIR-II excitation, especially at wavelengths greater than 1500 nm; with this reduction in auto-fluorescence contributing to enhanced spatio-temporal resolution and fidelity in 3D images (Li and Wang, 2018; Ding et al., 2018; Tian et al., 2020). The NIR-II window can be further sub-divided into two wavelength ranges spanning across a peak in water absorption at 1450 nm (Figure 1), i.e. the NIR-IIa (1000–1400 nm), and the NIR-IIb (1500–1800 nm) (Ma et al., 2021a; Feng et al., 2021). In spite of a higher endogenous absorption re-emerging at such wavelengths, several studies have revealed that the higher absorption coefficient of water can be beneficial in depleting the amount of scattered photons, thereby allowing “ballistic” photons to travel deeper into the tissue. This effect is known as “absorption-induced resolution enhancement” and can therefore produce clearer fluorescence images (Yoo et al., 1991; Sordillo et al., 2014b; Carr et al., 2018a; Feng et al., 2021).

The advancement from the NIR-I to the NIR-II optical window in fluorescence imaging has been facilitated by the development of NIR-II-absorbing probes suitable for biological imaging and by the increased availability of photodetectors sensitive enough in this spectral range (Hong et al., 2017). Currently, a variety of NIR-II imaging probes, including single-walled carbon nanotubes (SWCNTs), quantum dots (QDs), rare-earth doped nanoparticles (NPs), organic dyes, and semiconductor polymer NPs have been reported but these often have poor water solubility and limited physiological stability. Most significantly, as emitted photons are of lower energy than standard single-photon (1P) excitation, NIR-II-emitting fluorophores usually suffer from poor emission quantum yields (Φ_f_). Risks are also associated with the use of inorganic nano-systems including possible toxicity and lack of tissue specificity (Ding et al., 2018; Cao et al., 2019; Zhang et al., 2021a; Chen et al., 2021; Wang et al., 2021). The specific design of NIR-II-absorbing organic probes for bio-imaging has become a key challenge in the discipline, involving the multi-step synthesis of bulky water insoluble structures which often require complex purification (Sordillo et al., 2014a; Hong et al., 2017; Wanderi and Cui, 2022). On the other hand, a number of visible and NIR-I-absorbing fluorophores exhibiting high quantum yields are commercially available, and have the ability to target a wide range of biological substrates (Carr et al., 2018b). Such dyes are comparatively easy to synthesise and are routinely used for bio-imaging using one-photon absorption (1PA) (Escobedo et al., 2010). The use of two-photon (2P) fluorescence microscopy can facilitate the imaging in the NIR-II window by targeting 1P-absorbing visible/NIR-I dyes but with the added advantage of deep tissue penetration, exceptional feature clarity and high SBR (Carr et al., 2018b).

**FIGURE 2:**
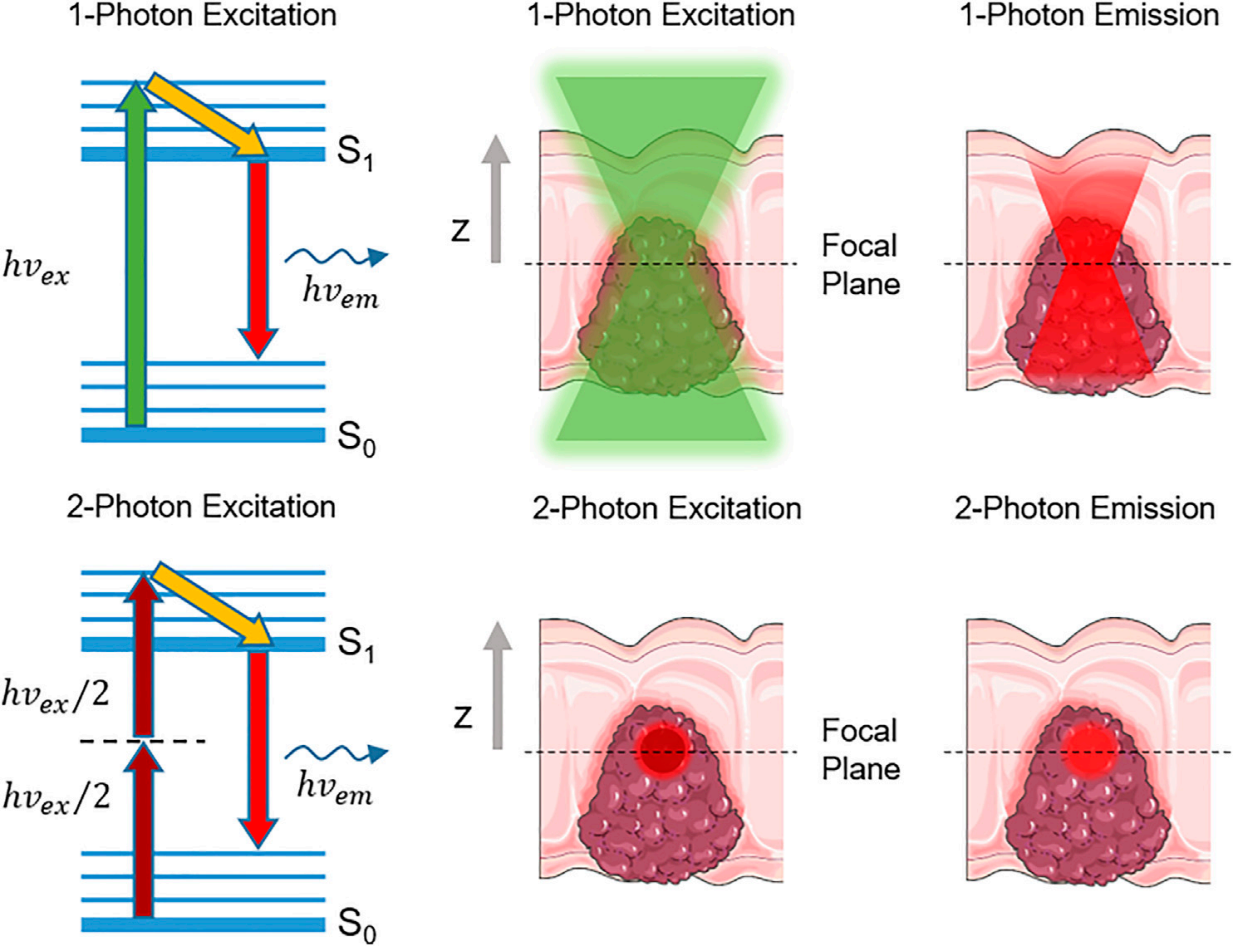
(Left) Simplified Jablonski diagram illustrating the double-photon absorption and single-photon emission involved in 1PA vs. 2PA excitation (Middle) Demonstrates the quadratic excitation that arises in 2PA vs. 1PA, due to the requirement of two photons having to arrive simultaneously at a sample to result in excitation (Right) The resulting tissue is capable of producing fluorescence emission due to the location of the excitation photons in 2PA vs. 1PA, resulting in more focused, high-resolution images in 2PA fluorescence imaging.

First predicted theoretically by Maria Göppert-Mayer in 1929 (Göppert, 1929; Göppert-Mayer, 1931), 2P absorption (2PA) is a third-order, resonant non-linear optical (NLO) process (Vivas et al., 2018; Ewart and Guenther, 2005) using the combined energy of two photons to generate an electronic transition from the ground state (S_0_) to a singlet excited state (S_*n*_) (Figure 2, left) (He et al., 2008; Pawlicki et al., 2009; Klausen and Blanchard-Desce, 2021; Pascal et al., 2021). Contrary to 1P excitation (1PE), 2P excitation (2PE) therefore requires near-simultaneous absorption of two photons of the same frequency ν (degenerate 2PA) or different frequencies ν_1_ and ν_2_ (non-degenerate 2PA). The excitation occurs as a two-step process, firstly involving a transition to a short-lived (sub-femtosecond) non-resonant excited state, called the “virtual state” (dashed line, Figure 2, left). Assuming that each chromophore is exposed to the same laser cross-section, photons must arrive on the attosecond timescale to further promote electron excitation to a singlet excited state. Furthermore, as with all non-linear processes, the relationship between the excitation light intensity and fluorescence intensity is non-linear (quadratic) and therefore excitation can only occur when the photon flux of the excitation light is in the range of 10^20^–10^30^ photons/(cm^2^·s) (Ewart and Guenther, 2005). This high energy density can be achieved by using an ultra-short (∼100 fs) pulsed (∼80 MHz) laser system (Ávila et al., 2019). In such non-linear conditions, the capacity of a dye to absorb 2P light differs from standard 1PE. The 2PA capacity of a dye is defined as its 2PA cross-section (e.g. the effective “photon-catching area” of the molecule), noted as σ_2_ and expressed in Göppert–Mayer unit (1 GM = 10^–50^ cm^4^·s·photon^−1^), as a tribute to Maria Göppert-Mayer’s work (He et al., 2008; Pawlicki et al., 2009; Klausen and Blanchard-Desce, 2021; Pascal et al., 2021).

After excitation and internal conversion, the electron relaxes to the lowest singlet excited state (Kasha’s rule), from where all radiative and non-radiative decays occur, regardless of the type of excitation. With regards to biomedical imaging applications, this is essential, as the fluorescence generated (emission wavelength and efficiency) during radiative decay is the same after either 1PE or 2PE (Denk et al., 1990; Ewart and Guenther, 2005; Benninger and Piston, 2013). The efficiency of the radiative decay process is quantified by the fluorescence quantum yield Φ_f_, representing the number of photons emitted per photon absorbed. In microscopy applications, the overall brightness of a fluorescent imaging agent at a given wavelength is therefore defined as the product of its absorption capacity (ε^(λ)^ in 1PA, or σ_2_^(λ)^ in 2PA) and its emission quantum yield Φ_f_. In 2P applications, the 2P brightness σ_2_Φ_f_ thus allows direct comparison between fluorophores (Kim and Cho, 2015).

**FIGURE 3:**
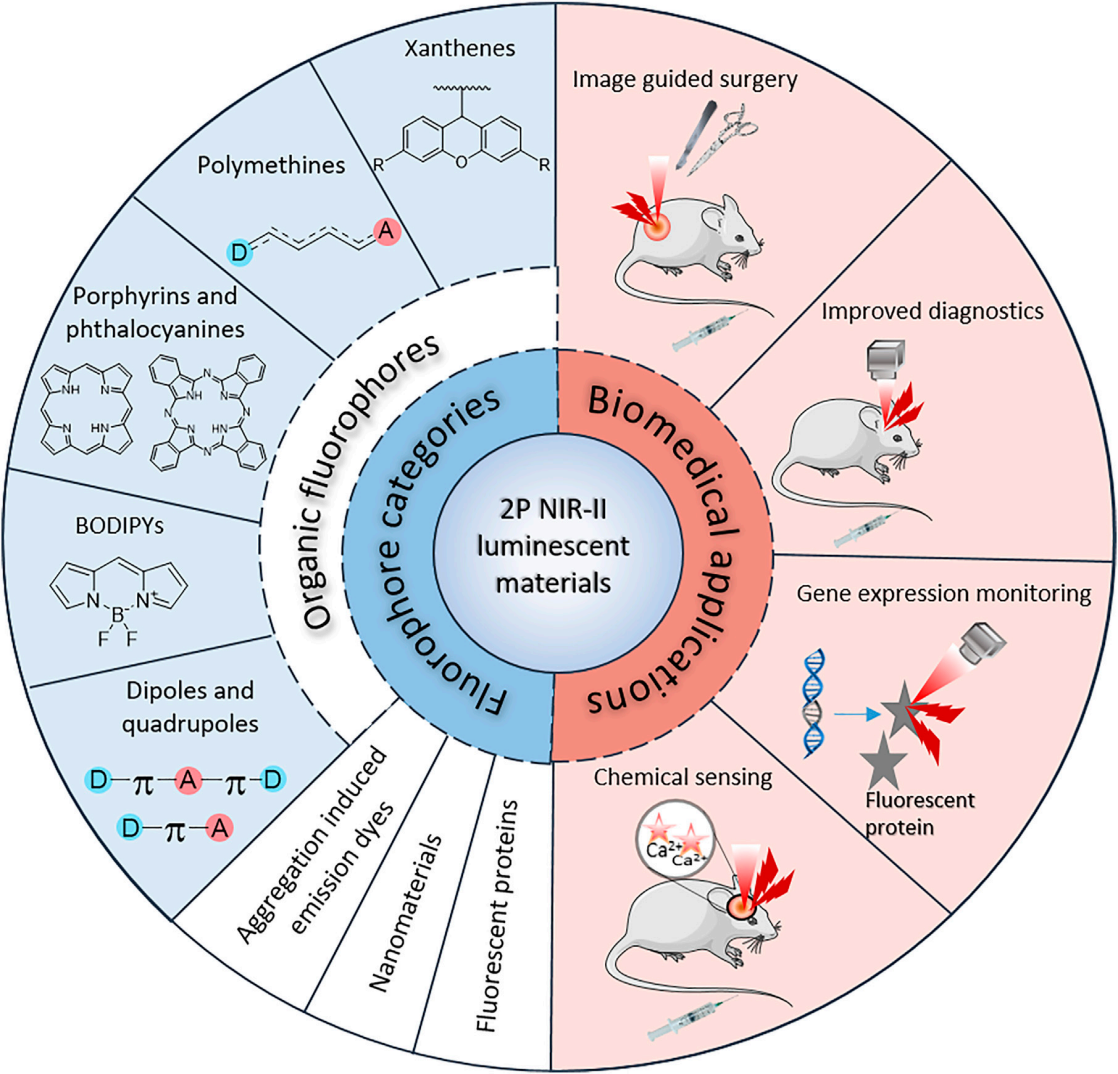
Overview of fluorophore categories and potential biomedical applications with 2PA NIR-II imaging.

Further adding to the imaging benefits, as the quadratic nature of the 2P process confines the excitation to a femtoliter-sized volume where the light intensity is the highest, 2PE avoids photon absorption and fluorescence both above and below the focal point (Figure 2, middle and right). As the fluorescence only originates from the focal point without out-of-focus emission of light, 2P microscopy provides inherent “confocality”, which allows high-resolution and high contrast imaging of thick living samples (Denk et al., 1990; Piston, 2005). This also prevents extensive photo-bleaching and photo-toxicity in live samples (Looney et al., 2011; Lu et al., 2020). Thanks to such unparalleled advantages over linear 1PE, 2PA has not only been extensively employed in bio-imaging and cell signal monitoring (Benninger and Piston, 2013; Kim and Cho, 2015; Kucikas et al., 2021; Helmchen and Denk, 2005; Ricard et al., 2018), but also in photodynamic therapy (PDT) (Sun et al., 2017a) and drug delivery (Klausen and Blanchard-Desce, 2021). In the context of NIR-II bio-imaging in particular, 2PE provides alternative solutions to the challenges met with standard 1PE. While the development of 1P-absorbing NIR-II-emitting OMI probes intrinsically leads to a high loss in brightness, 2PA directly exploits the emissive properties of 1P dyes at shorter wavelengths, which circumvents any loss of fluorescence quantum yield. The detection of fluorescence is also maximized with the use of common visible/NIR detectors with high sensitivities compared to their NIR-II counterparts, and by the increased distance between excitation and emission wavelengths preventing loss of signal due to spectral overlap. In addition, while standard single-photon confocal can only image samples of up to 200 μm in thickness, 2P microscopy improves imaging penetration depth by at least 2-fold relative to confocal imaging (Wang et al., 2017; Rubart, 2004). Several studies have also shown improved biocompatibility of 2P imaging as compared to 1P confocal imaging (Wokosin et al., 1996a). Squirrel *et al.* demonstrated that 8h of confocal imaging at 514 nm resulted in the inhibition of hamster embryo development (Squirrell et al., 1999), even without staining. In contrast, embryo viability was maintained when imaged using a 1047 nm ultrashort pulsed laser with the same microscope system for a 24-hour period. Importantly, similar experiments have also demonstrated that even NIR-I femtosecond irradiation could impair cell division at low power, and even lead to complete cell destruction (König et al., 1997). Therefore, the development of 2P microscopy in the NIR-II optical window represents an opportunity for higher-resolution and safer cellular imaging and would also facilitate a wider range of biological imaging applications such as image-guided surgery, diagnostics, gene expression monitoring, and chemical sensing (Figure 3). Such advanced applications have not yet been fully explored *in vivo* with NIR-II 2PA, but have shown great promise in *ex vivo* examples or utilizing shorter wavelengths for 2PE (Paoli et al., 2009; Grienberger and Konnerth, 2012; Cao et al., 2013; Fan et al., 2018).

In order to visualise, characterise and quantify biological entities, bright molecular imaging probes are needed (Yang et al., 2020a). To generate bright 2P microscopy images without causing considerable photo-damage to the sample at laser intensities required (1 GW·cm^−2^ at the focal plane; or ∼5 mW at the objective lens), it is estimated that the 2P brightness σ_2_Φ_f_ of the imaging agent should be more than 50 GM (Kim and Cho, 2015; Osmialowski et al., 2020). As such, breaking down the barriers to exploit NIR-II wavelengths in 2P bio-imaging involves three main challenges: (i) shifting the 2PA band of the imaging agent beyond 1000 nm, which typically involves 1PA above 500 nm; (ii) improving the 2PA cross-section σ_2_ above this wavelength, typically to values >50–100 GM; and (iii) retaining a high enough fluorescence quantum yield Φ_f_ to image tissues with high contrast. High water solubility, *in vivo-* and photo-stability, target specificity, and low toxicity are other general key criteria to develop ideal, clinically translatable OMI probes (Kim and Cho, 2015; Yang et al., 2020a; Rao et al., 2007; Yao and Belfield, 20122012). Small organic fluorophores (Wang et al., 2020a; Wu et al., 2022), aggregation induced emission (AIE) dyes (Lu et al., 2020; Zhu et al., 2018), inorganic and hybrid nanomaterials (Yao et al., 2014) and fluorescent proteins (FPs) are key types of materials that have been used in the development of OMI probes to date, and have shown high potential in the field of 2P in the NIR-II region (Figure 3). NIR-II-absorbing 2P-responsive dyes find applications in several additional areas beyond the scope of this review, such as optical power limiting (Pascal et al., 2021), chemical and ion sensing (Ricard et al., 2018), or targeted photo-therapies (Sun et al., 2017a; Zhao et al., 2019). In this review, we aim to present the current state of available luminescent 2P probes in a biological imaging and microscopy context, and highlight the recent progress and tremendous potential in this field. In the first subsection, we present the different classes of materials available for such applications and summarise their key optical properties in Table 1. We then present the available pulsed excitation sources used for such applications and discuss examples of 2P *in vivo* imaging in this “second optical window” by exploring imaging and lasing systems (Table 2), and tissue penetration depths (Table 3).

**TABLE 1:** 1PA, 2PA and emission properties of NIR-II chromophores reported in literature. Solvent and method of 2P properties are also noted for comparison.

Probe	Chemical structure	Solvent	λ_1PA_^max^	λ_em_^max^	Φ_f_	2λ_1PA_^max^	λ_2PA_	σ_2_^(λ)^	σ_2_^(λ)^Φ_f_	Ref.
			(nm)	(nm)		(nm)	(nm)	(GM)^a^	(GM)^a^	
**Xanthenes**										
Disodium fluorescein **(1)**	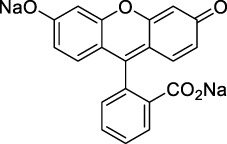	pH11	497	518^c^	0.90^c^	994	1000	2.7	2.4^b^	(Makarov et al., 2008; Mütze et al., 2012)
PhenGreen FL (diacetate, uncomplexed) **(2)**	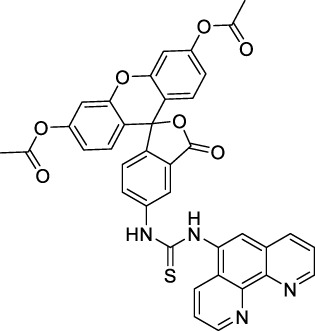	PBS	492^c^	517^c^	0.80^c^	984	1074	n.d.	n.d.	(Bestvater et al., 2002)
Rhodamine 6G **(3)**	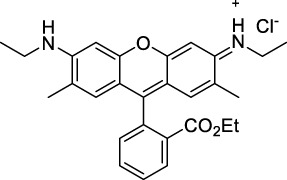	MeOH	519^c^	546^c^	0.95^c^	1038	1060	10	9.5^b^	(Makarov et al., 2008)
Rhodamine B **(4)**	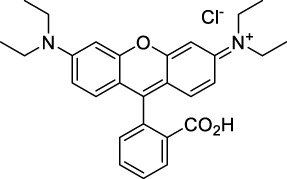	MeOH	553^c^	627^c^	0.70^c^	1106	1040	39	27^b^	Makarov et al., 2008
Rhodamine 101 **(5)**	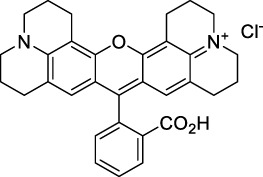	EtOH	570^c^	591^c^	1.0^c^	1140	1060	20	20^b^	(Li and She, 2010; Mütze et al., 2012)
Rhodamine 123 **(6)**	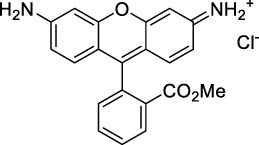	PBS	507	529^c^	0.90^c^ (EtOH)	1014	1090	n.d.	n.d.	(Bestvater et al., 2002)
Alexa Fluor 488 **(7)**	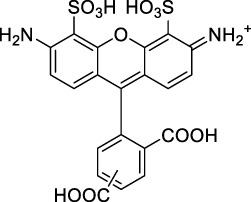	NaPhos	491	519^c^	0.92^c^	980	1000	21^b^	19	(Bestvater et al., 2002; Anderson and Webb, 2011; Mütze et al., 2012)
Alexa Fluor 546 **(8)**	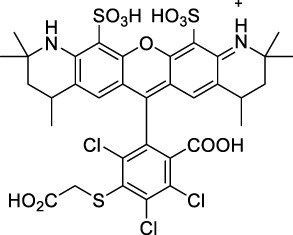	PBS	553	573^c^	0.79^c^	1112	1028	n.d.	n.d.	(Bestvater et al., 2002; Mütze et al., 2012)
Alexa Fluor 568 **(9)**	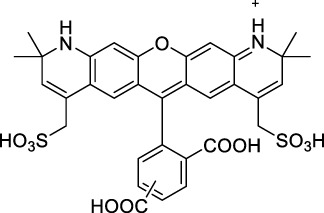	PBS	578^c^	603^c^	0.69^c^	1156	1060	n.d.	n.d.	(Mütze et al., 2012)
Alexa Fluor 594 **(10)**	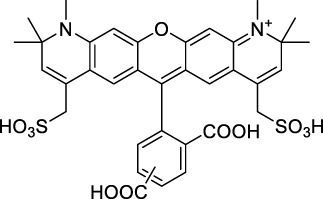	PBS	594^c^	617^c^	0.66^c^	1180	1074	n.d.	n.d.	(Bestvater et al., 2002; Mütze et al., 2012)
Alexa Fluor 610 **(11)**	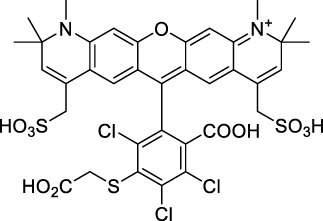	PBS	612^c^	628^c^	–	1224	1010	n.d.	n.d.	(Mütze et al., 2012)
Alexa Fluor 633 **(12)**	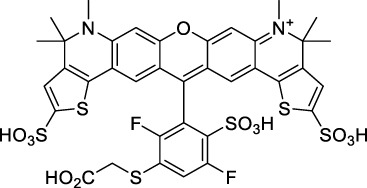	H_2_O	632^c^	647^c^	–	1264	1260	n.d.	<5	(Kobat et al., 2009; Mütze et al., 2012)
MitoTracker Red **(13)**	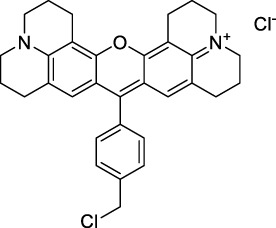	PBS	579^c^	599^c^	0.15 [187]	1158	1133	n.d.	n.d.	(Bestvater et al., 2002)
CellTracker Red **(14)**	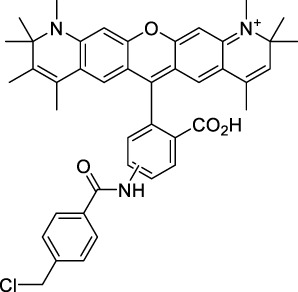	*In vitro*	585^c^	602^c^	n.d.	1170	1080	n.d.	n.d.	(Rakhymzhan et al., 2017)
Lissamine Rhodamine-IgG **(15)**	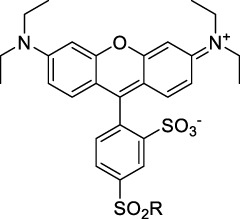	PBS	570^c^	590^c^	0.33 [188]	1140	1116	n.d.	n.d.	(Bestvater et al., 2002)
Texas Red-IgG **(16)**	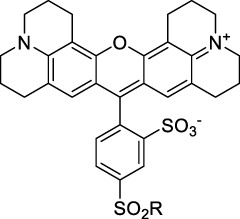	PBS	596^c^	615^c^	0.90^c^	1192	1150	n.d.	n.d.	(Bestvater et al., 2002)
ATTO 680 **(17)**	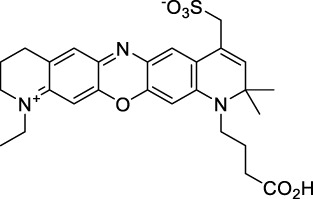	*In vitro*	681^c^	698^c^	0.30^c^	1362	1260	n.d.	n.d.	(Rakhymzhan et al., 2017)
Nile Red **(18)**	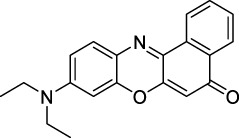	MeOH	550	636	0.40	1100	1057	104	42	(Hornum et al., 2020)
**19**	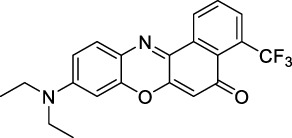	MeOH	554	631	0.43	1108	1055	183	79	(Hornum et al., 2020)
**20**	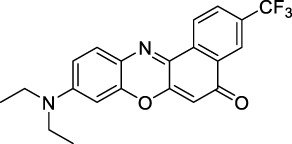	MeOH	569	632	0.45	1065	1050	123	55	(Hornum et al., 2020)
**21**	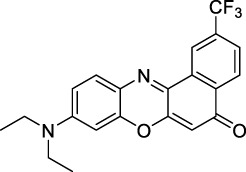	MeOH	565	638	0.35	1130	1057	232	81	(Hornum et al., 2020)
**Polymethines**										
Cy3-IgG (**22**)	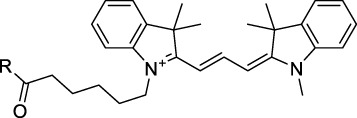	PBS	548^c^	563^c^	0.1^c^	1096	1032	n.d.	n.d.	(Bestvater et al., 2002)
Cy5 (**23**)	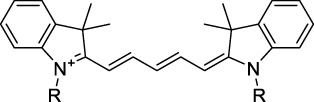	H_2_O	646^c^	662^c^	0.28^c^	1292	1220	143^b^	≈40	(Kobat et al., 2009)
Cy5.5 (**24**)	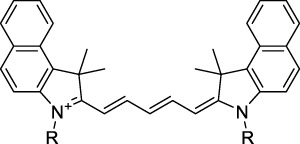	H_2_O	673^c^	691^c^	0.21^c^	1346	1280	286^b^	≈60	(Kobat et al., 2009)
Cy7 (**25**)	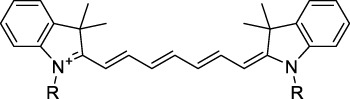	H_2_O	750^c^	773^c^	0.30^c^	1500	1320	200^b^	≈60	(Kobat et al., 2009)
**26**	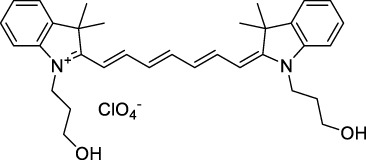	DMSO	753	780	0.17	1506	1552	240	41^b^	(Berezin et al., 2011)
ICG (**27**)	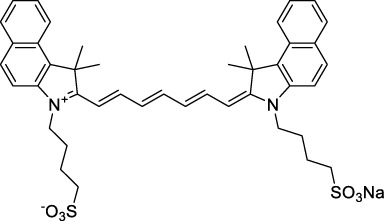	DMSO	794	817	0.12	1588	1552	590	71^b^	(Berezin et al., 2011)
Cypate (**28**)	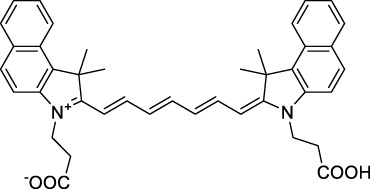	DMSO	796	817	0.13	1592	1552	520	68^b^	(Berezin et al., 2011)
**29**	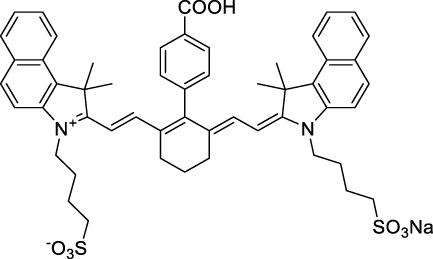	DMSO	809	829	0.07	1618	1552	900	63^b^	(Berezin et al., 2011)
DTTC (**30**)	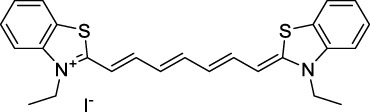	DMSO	771^c^	800^c^	0.80^c^	1542	1552	160	128^b^	(Berezin et al., 2011)
DODCI (**31**)	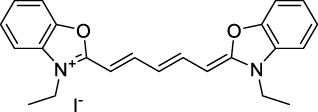	EtOH	582^c^	610^c^	0.87^c^ (DMSO)	1164	1060	38	n.d.	(Li and She, 2010)
IR-140 (**32**)	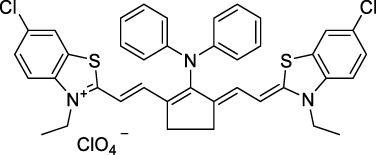	DMSO	825	≈840	0.06	1640	1552	950	57^b^	(Berezin et al., 2011)
**33**	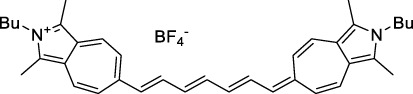	CH_2_Cl_2_	1064	≈1080	0.05	2128	1800	2250	113^b^	(Hu et al., 2013)
		CH_3_CN	1043	≈1065	0.05	2086	1800	1050	53^b^	
**34**	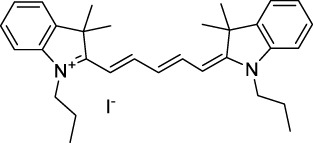	EtOH	650	665	n.d.	1300	1180	140	n.d.	(Fu et al., 2007)
**35**	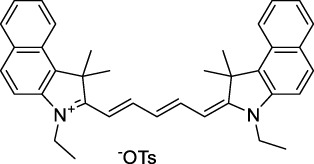	EtOH	690	704	n.d.	1380	1260	150	n.d.	(Fu et al., 2007)
**36**	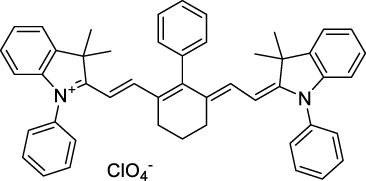	EtOH	770	n.d.	n.d.	1540	1340	60	n.d.	(Fu et al., 2007)
**37**	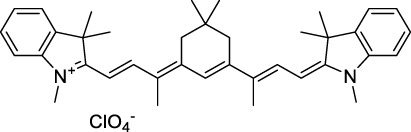	EtOH	824	n.d.	n.d.	1648	1480	600	n.d.	(Fu et al., 2007)
Alexa Fluor 647 (**38**)	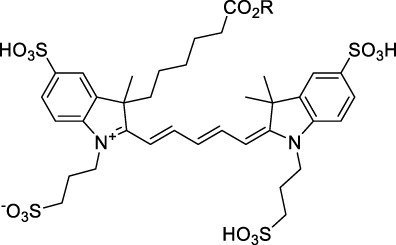	H_2_O	650^c^	665^c^	0.33^c^	1300	1240	133^b^	≈44	(Kobat et al., 2009; Mütze et al., 2012)
Alexa Fluor 680 (**39**)	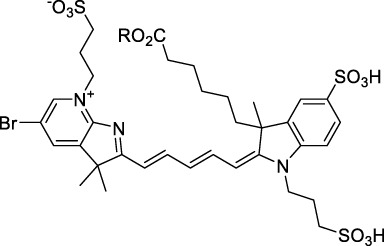	H_2_O	679^c^	702^c^	0.36^c^	1358	1280	203^b^	≈73	(Kobat et al., 2009)
Alexa Fluor 700 (**40**)	–^d^	H_2_O	702^c^	723^c^	0.25^c^	1404	1320	208^b^	≈52	(Kobat et al., 2009)
Alexa Fluor 750 (**41**)	–^d^	H_2_O	753^c^	778^c^	0.12^c^	1506	1320	292^b^	≈35	(Kobat et al., 2009)
**42**	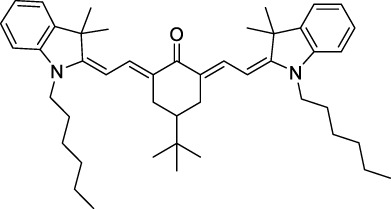	MeOH	532	636	0.44	1064	1064 (900)	23 (570)	10^b^	(Pascal et al., 2017)
**43**	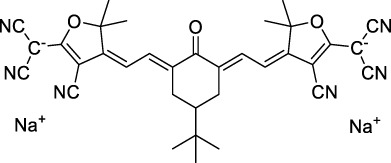	MeOH	573	708	0.33	1146	1146	225	74^b^	(Pascal et al., 2017)
**44**	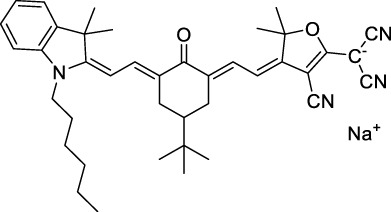	MeOH	549	673	0.54	1098	1098	137	74^b^	(Pascal et al., 2017)
**45**	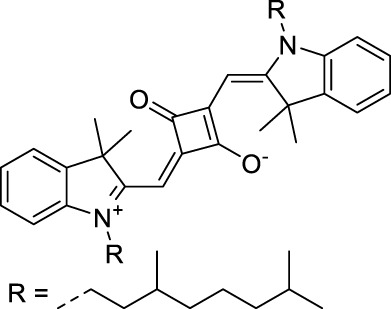	Toluene	643	654	0.62	1286	1198	133	82^b^	(Ceymann et al., 2016)
**46**	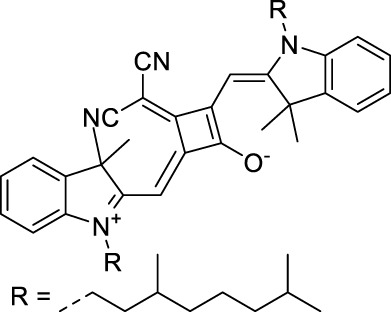	Toluene	700	714	0.75	1400	1274	100	75^b^	(Ceymann et al., 2016)
Styryl 9M (**47**)	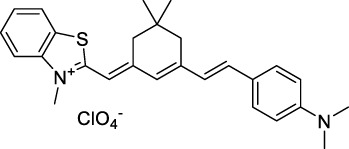	CHCl_3_	≈625	≈790	0.10 [189]	≈1250	1240	780	78	(Makarov et al., 2008)
FM4-64 (**48**)	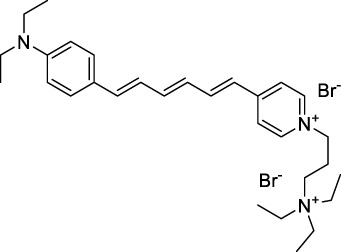	PBS CHCl_3_ (López-Duarte et al., 2015)	471 564	691 761	n.d. 0.35	942 1128	1047	n.d.	n.d.	(Wokosin et al., 1996a; Nuriya et al., 2016)
TO-PRO-3 (**49**)	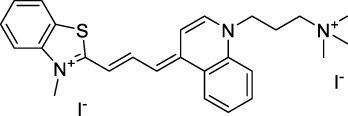	H_2_O	641^c^	657^c^	n.d.	1284	1110	n.d.	n.d.	(Smith et al., 2012)
**50**	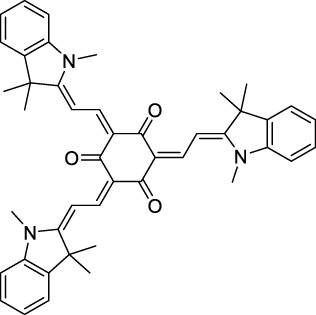	THF	562	598	0.07	1124	1070	167	12^b^	(Poronik et al., 2012)
**51**	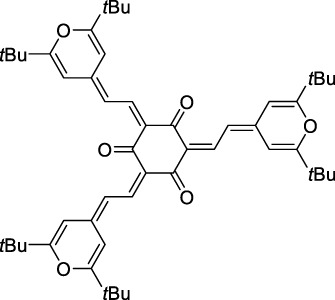	THF	615	655	0.02	1230	1150	214	4^b^	(Poronik et al., 2012)
**Porphyrins**										
**52**	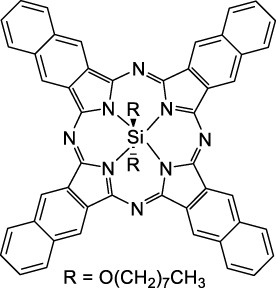	CCl_4_	≈770	≈780	n.d.	≈1540	1020 1270	470 48	n.d.	(Makarov et al., 2008)
**53**	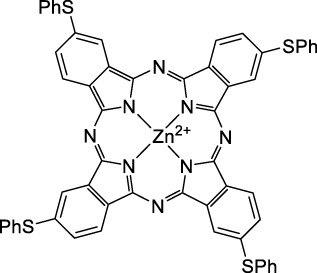	CCl_4_	≈685	≈700	n.d.	≈1370	1270	13	n.d.	(Makarov et al., 2008)
**54**	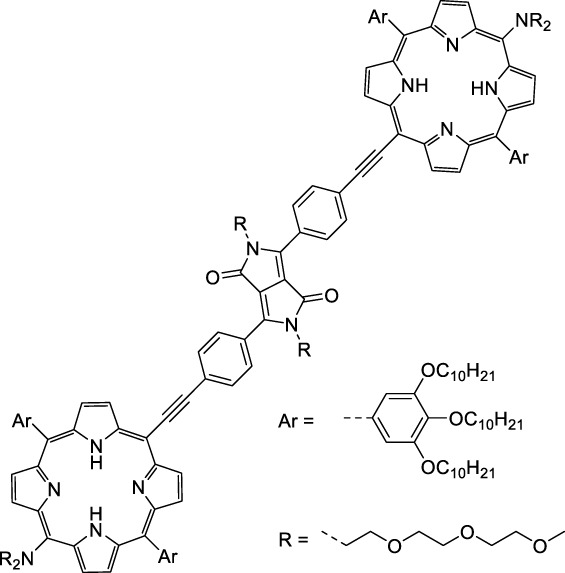	CHCl_3_	≈525 ≈605 ≈680	710	n.d.	≈1050 ≈1210 ≈1360	≈1040 ≈1220 ≈1360	≈2000 ≈500 ≈200	n.d.	(Nowak-Król et al., 2013)
**BODIPYs**										
LysoTracker Red (**55**)	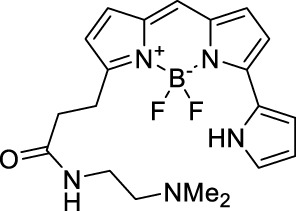	PBS	577^c^	590^c^	0.07	1154	1100	n.d.	n.d.	(Bestvater et al., 2002)
BODIPY-TR (**56**)	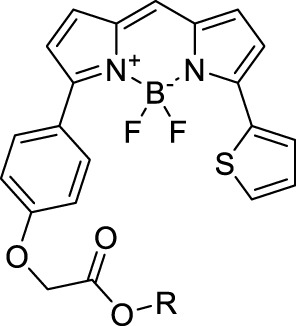	MOPS	589^c^	616^c^	0.90^c^	1178	1060	269^b^	242	(Bestvater et al., 2002; Mütze et al., 2012)
IR-07 (**57**)	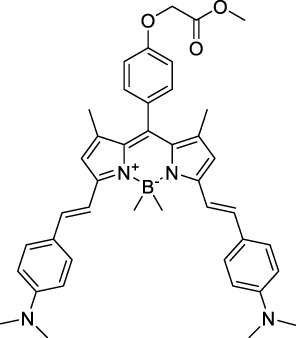	CH_2_Cl_2_	∼700	750	0.30	∼1400	1310	101	30^b^	(Zheng et al., 2009)
**58**	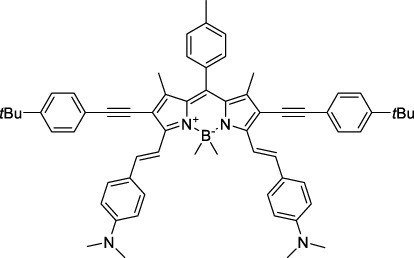	THF	755	830	0.09	1560	1064	n.d	n.d	(Hu et al., 2020)
**Dipoles – Quadrupoles – Miscellaneous**										
**59**	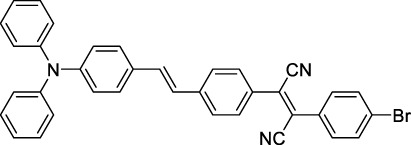	NPs (Aq.)	480	678	0.17	960	1040	5.6 × 10^5^	9520	(Alifu et al., 2017)
**60**	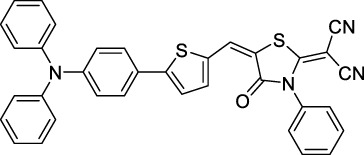	H_2_O (0.1% DMSO)	530	740	n.d.	1060	1100	n.d.	n.d.	(Zhou et al., 2021)
**61**	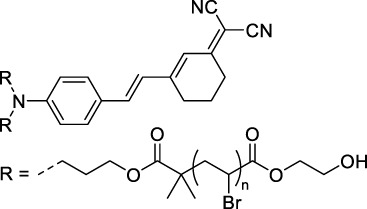	H_2_O	510	676	0.22	1020	1040	440	97^b^	(Massin et al., 2013)
**62**	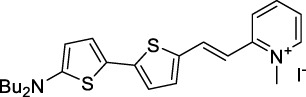	CH_2_Cl_2_	660	785	0.005	1320	1300	500	2.5^b^	(Ricci et al., 2017)
**63**	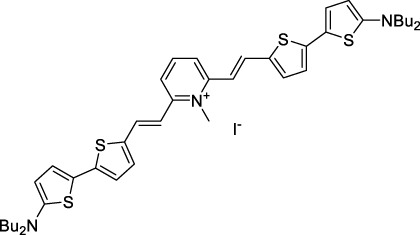	CH_2_Cl_2_	678	782	0.0005	1356	1300	1400	0.7^b^	(Ricci et al., 2017)
**64**	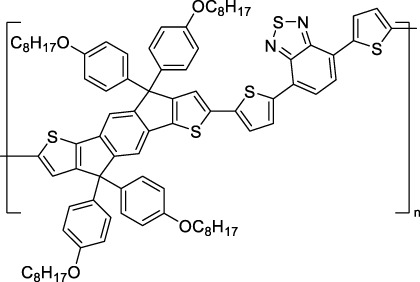	H_2_O	≈600	≈725	0.21	≈1200	1200	1.21 × 10^3^	242^b^	(Wang et al., 2019b)
**65**	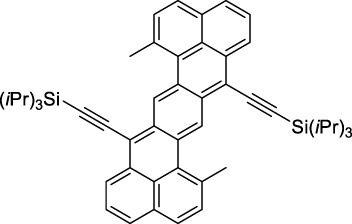	CHCl_3_	634	704	0.16	1268	1250	920	147^b^	(Li et al., 2012)
**66**	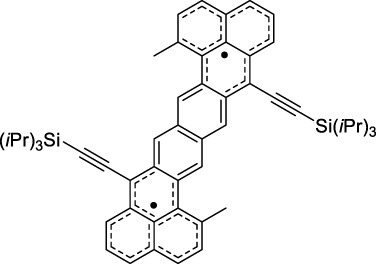	CHCl_3_	668	807	0.02	1336	1250	1200	24^b^	(Li et al., 2012)
**67**	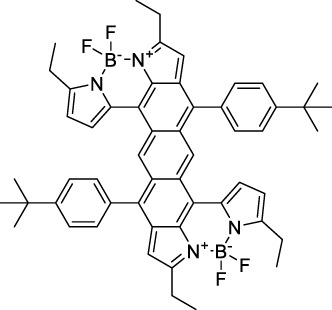	CHCl_3_	1088	1120	0.002	2176	2200	1300	2.6^b^	(Ni et al., 2016)
**68**	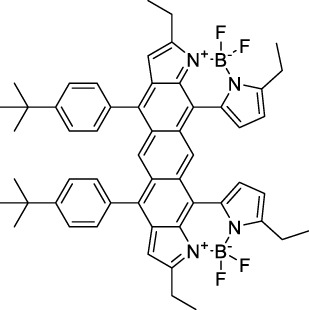	CHCl_3_	1136	1193	0.0002	2272	2300	1500	0.3^b^	(Ni et al., 2016)
Propidium iodide (**69**)	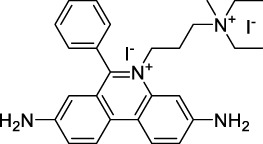	PBS	536^c^	617^c^	0.20^c^ (dsDNA bound)	1072	1015	n.d.	n.d.	(Bestvater et al., 2002)
**AIEgens and AIEDots**										
**70**	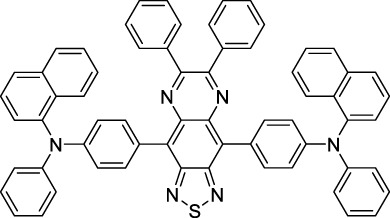	NP (aq.)	613	790-810	0.14	1226	1040 1300	16100 1220	2240^b^ 170^b^	(Qi et al., 2018; Liu et al., 2021)
**71**	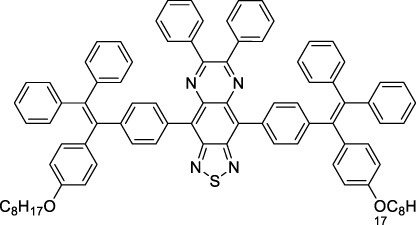	NP (aq.) THF	454 ≈451	≈700 ≈699	0.19 n.d.	908	1200 n.d.	76300 n.d.	14500^b^ n.d.	(Wang et al., 2019c)
**72**	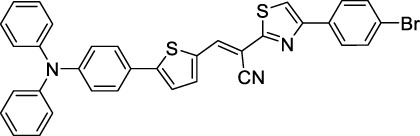	NP (aq.) H_2_O/DMSO	≈479 ≈488	≈627 >627	0.06 n.d.	≈960	1040 n.d.	3200 n.d.	192^b^ n.d.	(Samanta et al., 2021)
**73**	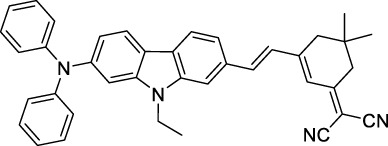	NP (aq.) Toluene	510 491	709 ≈635	0.14 (solid state)	1020	1000 n.d.	≈520 n.d.	73^b^ n.d.	(Zheng et al., 2018)
**74**	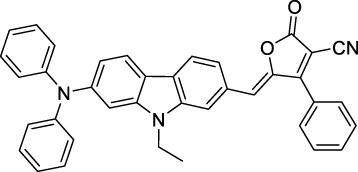	NP (aq.) Toluene	538 528	755 ≈636	0.02 (solid state)	1076	1020 n.d.	887 n.d.	18^b^ n.d.	(Zheng et al., 2018)
**75**	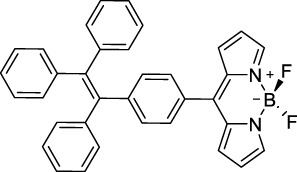	NP (aq.) THF	522 511	620 532	0.05n.d.	1044	1040 n.d.	2.9 ×10^6^n.d.	1.5 × 10^5^n.d.	(Wang et al., 2015)
**Carbon, hybrid and inorganic materials**										
**76**	SWCNT-based dopamine sensor	H_2_O	600–1000	1000–1265^e^	0.0023	–	1560	216000	497^b^	(Bonis-O'Donnell et al., 2017)
**77**	Aptamer-modified graphene oxide	H_2_O	440–720	500–650^f^	0.34	–	1120	36000	12240^b^	(Pramanik et al., 2014)
**78**	CDs prepared from urea and citric acid	H_2_O	540	624	0.06	1080	1200	n.d.	n.d.	(Li et al., 2018)
**79**	Carbon quantum dots prepared from tris(4-aminophenyl)amine	H_2_O	592	615	0.84	1184	1100	n.d.	n.d.	(Liu et al., 2020)
**80**	AuNP with SWCNT	H_2_O	500–1100	775	n.d.	–	1100	n.d.	n.d.	(Olesiak-Banska et al., 2019)
**81**	Au25 cluster	H_2_O	675	830	<0.001	1350	1290	2700	n.d.	(Ramakrishna et al., 2008)
**82**	PEG-dithiolane AuNC	H_2_O	355, 670	820	0.08	1370	1100	300	24^b^	(Oh et al., 2013)
**83**	Mn^2+^-ZnS QD	H_2_O	318	586	0.65	636	1180	265	172^b^	(Subha et al., 2013)
**84**	PbS/CdS QD	H_2_O	665	1270	0.18	1330	1550	530	95^b^	(Ni et al., 2022)
QD605 (**85**)	polymer-encapsulated CdSe-ZnS QD	H_2_O	350–475	605	0.71	–	1000	66200^b^	47000	(Larson et al., 2003)
**Fluorescent proteins**										
tdTomato (**86**)	–	H_2_O	554^g^	581	0.72^b^	1108	1050	278	200	(Drobizhev et al. 2011)
tdKatushka2 (**87**)	–	H_2_O	588^g^	633	0.44^b^	1176	1100	143	63	(Drobizhev et al. 2011)
dsRed2 (**88**)	–	H_2_O	561^g^	587	0.71^b^	1126	1050	103	73	(Drobizhev et al. 2011)
HcRFP (**89**)	–	PBS	592^g^	645^g^	0.05^g^	1184	1160	720^b^	36	(Tsai et al., 2006)
mCherry (**90**)	–	H_2_O	587^g^	610	0.24^b^	1174	1080	27	6.4	(Drobizhev et al. 2011)
mBanana (**91**)	–	H_2_O	540^g^	553	0.69^b^	1080	1070	64	44	(Drobizhev et al. 2011)
mStrawberry (**92**)	–	H_2_O	574^g^	596	0.34^b^	1148	1070	20	6.8	(Drobizhev et al. 2011)
mRFP (**93**)	–	H_2_O	584^g^	611	0.30^b^	1168	1080	44	13	(Drobizhev et al. 2011)
TagRFP (**94**)	–	H_2_O	555^g^	584	0.44^b^	1110	1050	95	42	(Drobizhev et al. 2011)
mOrange (**95**)	–	H_2_O	548^g^	565	0.70^b^	1096	1080	67	47	(Drobizhev et al. 2011)
eqFP650 (**96**)	–	H_2_O	592^g^	646	0.19^b^	1184	1112	45	8.5	(Drobizhev et al. 2011)
Katushka (**97**)	–	H_2_O	588^g^	635	0.35^b^	1176	1080	66	23	(Drobizhev et al. 2011)
Katushka2(**98**)	–	H_2_O	588^g^	633	0.44^b^	1176	1140	62	27	(Drobizhev et al. 2011)
mKate (**99**)	–	pH8	588^g^	635	0.27^b^	1176	1118	52	14	(Drobizhev et al. 2011)
mKate2 (**100**)	–	H_2_O	588^g^	633	0.42^b^	1176	1140	72	30	(Drobizhev et al. 2011)
mNeptune (**101**)	–	H_2_O	600^g^	651	0.17^b^	1200	1104	70	12	(Drobizhev et al. 2011)
mRaspberry (**102**)	–	H_2_O	598^g^	625	0.19^b^	1196	1118	31	5.8	(Drobizhev et al. 2011)
Neptune (**103**)	–	H_2_O	600^g^	647	0.22^b^	1200	1104	72	16	(Drobizhev et al. 2011)
tdRFP (**104**)	–	Aq. buffer	584 (Campbell et al., 2002)	579	0.68	1168	1110	20	13.7	(Drobizhev et al. 2011)

ab Two-photon absorption cross-section value taken at the excitation wavelength λ_2PA_ reported by the authors in the NIR-II window; note that this may differ from the maximum of the 2PA band. Value extrapolated from the data available and from the formula of the 2P brightness (= σ_2_^(λ)^ × Φ_F_).

c 1P properties as reported by commercial suppliers, reported in water unless indicated otherwise.

d Chemical structures are propriety and undisclosed.

e Chirality-dependant.

f Excitation-dependant.

g Properties extracted from the fluorescent protein database (Available at https://www.fpbase.org/, Accessed on 11/04/2022).

**TABLE 2:** Benefits associated with Fibre lasers over traditional solid-state and OPO lasers (adapted from ref (RPMC, 2020))

Solid-State/OPO-Based Lasers	Femtosecond Fibre Lasers
Large cumbersome design	Lightweight, up to 10x smaller
Expensive	Affordable
Less consistent pulse duration	Pulse duration always maintained
Lower power at long wavelengths (crystal-based amplifiers)	Maintains high power at long wavelengths (up to 4W at 920 nm)
Water-cooled	Air-cooled
High cost of ownership	Low maintenance and engineer fees (fast return on investment potential)

**TABLE 3:** Current examples of 2PA *in vivo* experiments performed using NIR-II absorbing fluorophores.

λ_2PA_ (nm)	Imaging System	Laser used	λ_em_ (nm)	Contrast media	Depth (µm)	Animal model	Biological media imaged	Administration Method	Toxic. Test *in vivo* (Y/N)	Year	Ref
1550	Home-built system	PolarOnyx Mercury **Fibre laser**	800	DTTC dye (**30**)	180	Mouse	Resected kidney vasculature	Intravenous injection	N	2011	Berezin et al. (2011)
1200	Commercial Leica TCS SP8 MP	Laser with **OPO**	630	Squaraine **45**	40	Mouse	Ear vasculature	Ear blood vessel injection	N	2019	Yi et al. (2019)
1057	Home-built with BioRad MRC 600 confocal microscope	Nd:YLF **solid state laser**	∼590	FM4-64 dye (**48**)	45	Zebrafish embryo	Body	Pre-stained	N	1996	Wokosin et al. (1996a)
1040	Home built with Olympus, BX61 FV1200 microscope	Yb-doped photonic crystal **fibre laser**	∼700	Triphenylamine dipole **59** encapsulated in PSMA NPs	1200	Mouse	Ear vasculature Brain vasculature Resected tumour vasculature	Tail vein injection	Y	2017	Alifu et al. (2017)
1100	Commercial Olympus FVMPE-RS	InSight® DS with **OPO**	740	PDT-imaging agent 60	210	Mouse	Resected tumour vasculature	Intravenous injection	Y	2021	Zhou et al. (2021)
1200	Commercial Olympus FVMPE-RS	InSight® DS with **OPO**	∼700	BTZ SNPs (**64**)	1010	Mouse	Brain vasculature	Rectoorbital injection	N	2019	Wang et al. (2019b)
1300	1. Home built with Olympus, BX61W1- FV1200 microscope 2. Home-Built system	1. PHAROS-10W with **OPA** 2. Laser from an **OPA**	810	AIEDots of BTZ dye **70** (∼35 nm)	1065	Mouse	Ear vasculature Brain vasculature	Tail vein injection	Y	2018	Qi et al. (2018)
1040	Home built with Olympus, BX61 FV1200 microscope	Mira HP and Mira **OPA**	790	AIEDots of BTZ dye **70**	750	Mouse	Brain vasculature	Tail vein injection	N	2021	Liu et al. (2021)
1200	Commercial Olympus FVMPE-RS	InSight® DS with **OPO**	∼700	AIEDots of BTZ dye **71**	800	Mouse	Brain/Ear tumour vasculature	Rectoorbital injection	Y	2019	Wang et al. (2019c)
1040	Nikon A1 Confocal Microscope	Laser with **OPO**	∼650	AIEDots of triphenylamine dye **72**	800	Mouse	Brain vasculature	Retro orbital injection	Y	2021	Samanta et al. (2021)
1040	Home built with Olympus, BX61W1-FV1000	Yb-doped photonic crystal **fibre laser**	∼620	AIEDots of BODIPY dye **75**	700	Mouse	Brain vasculature	Intravenous injection	Y	2015	Wang et al. (2015)
1100	Commercial Leica SP8 DIVE	Laser with **OPO**	615	Carbon quantum dots **79**	500	1. Zebrafish larvae 2. Mouse	1. Zebrafish Body (2PA) 2. Mouse (1PA)	1. Dots added to media 2. Intravenous injection	1. N 2. Y	2020	Liu et al. (2020)
1550	Home built with Olympus, BX61 FV1200 microscope	FLCPA-01C Calmar **fibre laser**	1270	PbS/CdS quantum dots **84**	220		Brain vasculature	Intravenous injection	N	2022	Ni et al. (2022)
1070	Home built system	Ti:Sapphire laser with **OPO**	∼580	Red Fluorescent Protein (tdTomato **86**)	300–500	Mouse	Brain neurons	Gene Expression (Six3 gene)	N	2013	Yang et al. (2013)

## 2 NIR-II-Absorbing Luminescent Materials for 2P Bio-Imaging

### 2.1 Organic Fluorophores

Reaching the NIR-II window with 2P excitation typically involves using dyes with 1PA maxima ranging from orange to NIR-I wavelengths. The design of 2P-responsive organic fluorophores has been covered extensively in several reviews and falls beyond the immediate scope of this review (He et al., 2008; Pawlicki et al., 2009; Klausen and Blanchard-Desce, 2021; Pascal et al., 2021; Kim and Cho, 2015). However, to achieve such results the following parameters must be taken into account. Even more so than in the context of standard 1PE, the size/length of the π-conjugated system and the magnitude of intramolecular charge transfer (ICT) are major driving forces for the 2PA capacity of a chromophore. Therefore, selecting strong electron-donating (ED) and electron-withdrawing (EW) moieties or extending the π-conjugated backbone in a push–pull compound are typical strategies to cause both ICT-induced bathochromic shifts in absorption wavelengths and increase in 2PA cross-sections. Nonetheless, to achieve NIR-II absorption, the selection rules of 2PA should be considered due to their direct effect on allowed electronic transitions within the molecule, which in turn affects its maximum absorption wavelength. Symmetry-based selection rules state that 2P electronic transitions at 2 × λ_1PA_^max^ wavelengths are forbidden in centrosymmetric chromophores. As a result, 2PA bands in symmetrical dyes are usually more intense, but shifted to higher energies, which can be a limitation in the design of NIR-II-absorbing dyes. Dipolar dyes present no such restriction as the transition to the first excited state is generally both 1P- and 2P-allowed. Therefore, 2P-absorbers with dipolar (D-π-A) or symmetrical (quadrupolar D-π-A-π-D, A-π-D-π-A; or octupolar D-(π-A)_3_, A-(π-D)_3_) structures, sometimes belonging to well-known classes of dyes, have been investigated in recent years and will be reviewed below. With the development and increased accessibility of SWIR pulsed laser technologies (Section 3, Tables 2 and 3), several studies have shown that remarkable potential resides in the pool of current biological stains and FDA approved fluorophores that offer such 1PA properties (Wokosin et al., 1996b; Bestvater et al., 2002; Fu et al., 2007; Makarov et al., 2008; Kobat et al., 2009) (Table 1).

#### 2.1.1 Xanthene Derivatives

Thanks to their exceptional structural diversity, xanthene-type dyes are among the most widely used biological imaging agents. The highly versatile xanthene backbone allows for extensive structural modification, with the possibility to induce dramatic bathochromic shifts in absorption and emission through dye design. As a result, the 1PE bands of such fluorophores can range from green to NIR-II wavelengths (Liu and Scott, 2021), leading to the majority of current research being focused on the design of NIR-I to NIR-II 1P-absorbing xanthene dyes. To the best of our knowledge, only a small number of xanthene dyes have been specifically designed for 2PA in the NIR-II, but the vast number of commercially available probes in this family facilitates their use as a model for 2P measurements. This in turn has led to several seminal reports on their 2P properties at NIR-II wavelengths since the first examples in 1972 (Bradley et al., 1972).

The 2PA properties of several commercial fluorescein- and rhodamine-based probes have been studied thoroughly and are well reported (Bestvater et al., 2002; Makarov et al., 2008; Bradley et al., 1972; Li and She, 2010; Hermann and Ducuing, 1972). Fluoresceins, being the most blue-shifted xanthene dyes, possess almost no 2P response in the NIR-II window (Table 1), as evidenced by the negligible σ_2_ of disodium fluorescein (**1**) at 1060 nm (0.069 GM) (Makarov et al., 2008). However, the heavy metal indicator PhenGreen FL^TM^ (**2**), a fluorescein derivative, was reported to show fluorescence when excited under 2PA at 1074 nm (Bestvater et al., 2002). Thanks to their higher internal charge transfer (ICT) and superior structural variety, Rhodamines possess a higher potential for 2PE in the NIR-II. Rhodamines 6G (**3**), B (**4**), 101 (**5**) and 123 (**6**) all present 2PA beyond 1000 nm (Table 1). Among them, Rhodamine B was reported with the highest 2PA cross-section in the early NIR-II window, with a second 2PA band at 1040 nm in MeOH (38 GM) (Makarov et al., 2008). Highly photo-stable Alexa Fluor^TM^ dyes 488 to 633 (**7–12**) were also reported with 2PA bands between 985 nm and 1264 nm respectively (Bestvater et al., 2002; Kobat et al., 2009; Anderson and Webb, 2011; Mütze et al., 2012). Alexa Fluor^TM^ 488 (**7**) responded moderately to 2PE at 1000 nm (Anderson and Webb, 2011), which corresponds to the tail of its absorption band. No quantification was performed on the other rhodamine-type Alexa Fluor^TM^ dyes, however, several other accessible cell-labelling and bioconjugatable rhodamine derivatives were investigated, such as MitoTracker Red (**13**), or Lissamine Rhodamine (**15**) and TexasRed (**16**) conjugates (Bestvater et al., 2002).

Phenoxazines are nitrogen-containing xanthene derivatives that also demonstrated 2PA properties in the NIR-II. Rakhymzhan *et al.* demonstrated the use of extended phenoxazine ATTO680 (**17**) in live cells under 2PE at 1260 nm (Rakhymzhan et al., 2017). Nile Red (**18**) is another well-known member of this class of dyes, and is commonly used as a reference for cross-section measurements using the 2P-excited fluorescence (2PEF) technique. In an effort to optimise the 2PA properties of Nile Red, Hornum *et al.* prepared and optically characterised a nu mber of halo-substituted Nile Red derivatives (Hornum et al., 2020). On this occasion, they measured a σ_2_ of 104 GM for Nile Red at 1057 nm. The σ_2_ of the reported derivatives were also measured between 1000 and 1200 nm, showing a substantial increase upon introduction of a trifluoromethyl group (compounds **19**, **20** and **21**). Interestingly, regio-isomer 2- (**21**) showed the highest gain in σ_2_ compared to isomers 3- and 4- (**19** and **20**). Nile Red derivatives are notably solvatochromic, which increased both their 2PA wavelengths and cross-sections by up to 313% with increasing polarity.

#### 2.1.2 Polymethine Derivatives

Polymethine dyes consist of an alternating single and double-bond backbone, which connects two aromatic moieties. Cyanine (Cy) dyes are a member of this family of dyes, with the polymethine conjugated chain separating two nitrogen-containing heterocycles (i.e. indolenine, benzindole). These core structures can tolerate numerous structural changes including: i) lateral groups (i.e. sulfo, carboxyl) on the heterocycles to increase solubility; ii) cycloalkane-rings within longer polymethine chains (Cy7, Cy7.5, squarines) for structural rigidity and photo-property tuning; iii) flexible chains terminating in functional groups (i.e. carboxylic acid, alkyne, azide, NHS ester) for attachment to drug probes (Lee et al., 2008; Pham et al., 2008; Henary et al., 2009; Sun et al., 2019). The most recently reported NIR-II polymethines have been designed for 1P-excited fluorescence (1PEF), including BTC1070 which employed a pentamethine chain, benzothiopyrylium heterocycles and diethylamino ED moieties to achieve emission above 1000 nm (Wang et al., 2019a). Selection rules will affect differently polymethines bearing identical or different substituents on each side of the poly-ene chain, which will therefore modify the nature of their dominant 2P transition. Largely blue-shifted transitions can be observed in certain environments in the case of symmetrical polymethines.

The polymethine family includes several commercially available and FDA-approved derivatives that have been evaluated under 2PE (Table 1). In 2002, Bestvater *et al.* reported the 2PA spectrum of Cy3 (**22**), showing a response at 1032 nm (Bestvater et al., 2002), and modest 2P brightnesses were then measured by Fu *et al.* and Kobat *et al.* for the original Cy5 (**23**), Cy5.5 (**24**) and Cy7 (**25**) fluorophores in the 1200–1300 nm range (Fu et al., 2007; Kobat et al., 2009). While there is much reported about the structure-1P property relationships in custom-made polymethine dyes, even towards the NIR-I and -II regime, less is known about their 2P properties. Berezin *et al.* studied the 2P properties of several cyanine derivatives with comparable conjugation under 1552 nm excitation, by varying the central and hetero-aromatic moieties (Lee et al., 2008; Berezin et al., 2011). Strong absorption was reported at this wavelength for compound **26**, a direct Cy7 analogue (240 GM).

Replacing the indolenine units with π-extended benzoindolenines causes an increase in ICT and oscillator strength leading to red-shifted optical properties and higher σ_2_. This is evidenced by the commercially available and FDA-approved dye indocyanine green (ICG, **27**) whose σ_2_ is more than doubled compared to **26**. Interestingly, ICG (**27**) was also used as a contrast agent for 2P fluorescence imaging at 790 nm (Kumari and Gupta, 2019), which leads to excitation in its blue-shifted S_0_→S_2_ band. The fluorophore thus presented an Anti-Kasha fluorescence at 570 nm, emitting directly from the S_2_ excited state, which provided it with an excitation-dependent 2PEF. ICG is now used in a clinical context for diagnostic purposes (Schaafsma et al., 2011; Hackethal et al., 2018), and therefore possesses a strong potential to develop 2P imaging past 1500 nm. Cypate (**28**), a bio-conjugatable version of ICG replacing both side sulfonate groups with carboxylic acids was also prepared and characterised, leading to similar optical properties (Berezin et al., 2011). Restriction of the polymethine chain with a phenylcyclohexene moiety (**29**) led to a 70% increase in cross-section but similar brightness. DTTC and DODCI (**30** and **31**), two benzothiazolyl and benzoxazolyl analogues of Cy7 and Cy5, were also used in studies at 1552 nm (Berezin et al., 2011) and 1060 nm (Li and She, 2010) respectively. The reported cross-section for DODCI was measured at a wavelength significantly different from the 2λ_1PA_ value, but was still considerably lower than for DTTC which contains an extra double bond in its π-conjugated system. Introducing aromatic units at the 4-position of the heptamethine chain led to increased cross-sections, but halved the quantum yield. IR-140 (**32**) therefore has the largest cross-section in this class of dyes thanks to a diphenylamine unit attached to the central cyclopentene-heptamethine chain; however, this does not yield a high brightness due to a poor Φ_f_. In this study, the best 2P brightness was calculated for DTTC, which was then selected for *ex vivo* 2PEF imaging of kidney tissue (Section 3) (Berezin et al., 2011). Additionally, the 2P properties of 2-azaazulene polymethine dye **33** were extensively studied theoretically and experimentally to elucidate their symmetrical character (Hu et al., 2013). By comparing the 2PA spectra of **33** in apolar dichloromethane and polar acetonitrile, the authors demonstrated a symmetry-breaking character in high polarity media leading to a restriction lifting of their forbidden transition at 2λ_1PA_^max^, which is a crucial observation for the development of NIR-II responsive polymethines. A set of structure-property relationships was also constructed by Fu *et al.* in a seminal report on the 2PA properties of polymethine dyes (Fu et al., 2007). Cross-sections ranging from 60 to 600 GM were measured in the NIR-II on different extended, locked and substituted cyanines (dyes **34–37**), which is consistent with other reports on similar dyes. This demonstrated similar effects of conjugation lengths and ICT on the 2PA spectra and cross-sections. A strong increase in σ_2_ was noted in particular for cyanine **37**, which presented the most constrained conformation. Alexa Fluor^TM^ 647 and 680 (**38–39**) are other commercially available polymethine analogues reported with moderate 2P brightnesses (Kobat et al., 2009; Mütze et al., 2012).

Adding ketones to the central polymethine chain was found to produce fluorescent dyes **42–44** that show significant red-shifting and quantum yield increase in protic solvents (Pascal et al., 2017). Because of their pseudo-quadrupolar character, these dyes exhibit strong blue-shifted 2PA bands in the NIR-I, with σ_2_ values ranging from 570 to 1400 GM at 900–970 nm. However, their transition at 2λ_1PA_ remains partially allowed, which leads to a second weaker 2PA peak in the 1100 nm region, reaching 250 GM in the case of bis-acceptor dye **44**. These dyes were modified to include hydrophilic, hydrophobic and water-solubilizing polymers, and were successfully used in 1P and 2P microscopy.

Squaraines are a particular example of keto-polymethine dyes combining two ED groups connected to a four strongly electron-deficient 4-membered ring system derived from squaric acid. This class of dyes is known for their potential to reach considerable 2PA cross-section values with relatively simple structures, which provides them with a high σ_2_ to molecular weight ratio (Chung et al., 2006; Sun et al., 2017b). Only a few examples of squaraine fluorophores were investigated in the NIR-II range; yet the simplest examples of squaraines, built from indolenine subunits, show intense 2PEF upon excitation past 1000 nm (Ceymann et al., 2016). Squaraine **45** and its malononitrile derivative **46** were described as bright red/NIR fluorophores (λ_em_ = 654 nm and 714 nm), with quantum yields of 0.62 and 0.75 respectively. Both dyes showed moderate 2P response (σ_2_ > 100 GM) around 1250 nm. Compound **45** was used for *in vitro* and *in vivo* for 2P imaging at 1200 nm (Yi et al., 2019). The authors demonstrated that the 2PEF of this small dye was enhanced 17.7 times in the presence of bovine serum albumin (BSA). Moreover, squaraine showed excellent photostability and low cytotoxicity. Interestingly, more advanced squaraine oligomers and branched structures were also investigated, showing high 2PA in the NIR-II (Scherer et al., 2002; Ceymann et al., 2016).

Other heptamethine cyanine dyes carrying different terminal heteroaromatic moieties (benzoindolenine, thiazole, oxazoles, azaindoles, flavyliums) have been developed and widely used as 1P contrast agents. Dimethylamino flavylium polymethine dyes have been shown to exhibit significant bathochromic shifts compared to their analogous Cy dyes, thus taking their 1PE up to 1026 nm (Cosco et al., 2017). Funabiki *et al.* also showed the importance of the counter-ion in benzo [cd]indolenyl-substituted heptamethine cyanine dyes (Funabiki et al., 2019). Already reaching the NIR-II window for *in vivo* 1PEF microscopy, these would be excellent candidates to have their 2P properties investigated.

A common trait of cyanine-type dyes is their pseudo-centrosymmetric character that can make the 2PA transition at 2λ_1PA_ partially forbidden (Hu et al., 2013), and therefore reduce their 2P brightness in the NIR-II. Non-symmetrical polymethines are another important sub-class of dyes that adopt a dipolar character and therefore overcome this feature. The 2PA spectrum of the commercially available dye Styryl 9M (**47**) was reported by Makarov *et al.*, which highlighted a high 2PA cross-section (750 GM), in the 1150–1250 nm region (Makarov et al., 2008). Styryl 9M was notably used for the detection of lysozyme amyloid fibrils with 2PE (Udayan et al., 2020). The nonpolar and viscous environment generated by the hydrophobic channels of lysozyme fibrils led to a strong bathochromic shift in the absorption spectrum of the dye, accompanied by an increase in quantum yield. FM4-64 (**48**) (Wokosin et al., 1996a) and To-Pro-3 (**49**) (Smith et al., 2012), two other non-symmetrical dipolar polymethines, were reportedly used in 2P imaging past 1064 nm. FM4-64 is also commonly used in second-harmonic generation experiments, which makes it a multi-modal imaging agent (Nuriya et al., 2016).

Merocyanines are a sub-group of dipolar polymethine chromophores constituted specifically of an amine (D) and a carbonyl (A) moiety, connected to each end of the poly-ene π-conjugated system. Merocyanines are typically sensitive to their local environment, with optical properties varying in contact with cell membranes, metal ions, or DNA; and 2PE was shown to be even more sensitive than 1PE to such variations in the local environment (Pascal et al., 2017). Fewer examples of 2P-responsive merocyanines have been reported in the NIR-II. However, in their investigation of keto-polymethines, Pascal *et al.* also reported a merocyanine-like dye **43** that showed a 2PA maximum at 1098 nm. Its moderate cross-section was compensated by a good Φ_f_, which led to a brightness value of 74 GM in MeOH. An interesting example of advanced merocyanine design was also achieved by incorporating the polymethine system onto a cyclohexanetrione moiety (Poronik et al., 2012). The resulting octupolar (D-π)_3_-A structures **50** and **51** showed moderate 2PA response in the NIR-II transition ranging between 98 and 214 GM in THF.

#### 2.1.3 Porphyrin and Phthalocyanine Derivatives

Porphyrins, phthalocyanines and other types of polypyrrole derivatives are commonly used as contrast agents or photosensitizers in biomedical applications (Josefsen and Boyle, 2012). The particular properties of such compounds make it possible to tune multiple parameters such as the lifetime of the excited state, and therefore their emissive character, by metalation. The 2PA properties of Zn-tetrakis-(phenylthio)-phthalocyanine (**52**) and Si-naphthalocyanine dioctyloxide (**53**), both commercially available, were investigated by Makarov *et al.* (Makarov et al., 2008). Both dyes showed 2PA bands at 1270 nm in CCl_4_ with moderate cross-sections (Table 1). The silicon derivative however showed significant 2PA capacity in its higher energy band (470 GM at 1020 nm). In contrast, tetraphenylporphyrin showed virtually no absorption past 1000 nm, which is in accordance with the limited 1PA capacity associated with its red Q-band. Porphyrin derivatives can however exhibit large cross-sections, as fused-systems show an increase in both cross-section and λ_ex2PA_ with the increasing number of rings (Yoon et al., 2007), although this can lead to preferential non-radiative behaviour. Only a few examples of polypyrrole design have led to NIR-II responsive fluorescent dyes specifically for 2P imaging applications. *Meso* substitution is nonetheless a typical design strategy to amplify the 2P response of porphyrin dyes (Nowak-Król et al., 2012), and porphyrin dimers bridged by a diketopyrrolopyrrole unit at this position were prepared to form highly absorbing D-π-A-π-D dyes (Nowak-Król et al., 2013). Although no quantification was performed in this study, the authors claim that their porphyrin dimer (**54**) remained fluorescent at 710 nm. A band structure characteristic of porphyrin dyes was observed on the 2PA spectrum, which leads to broad absorption between 1000 and 1450 nm. However, while these structures are of interest, their ability to be utilised in a bio-medical setting is limited due to poor solubility in aqueous environments, and the requirement of controlled self-assembly *in vitro*.

#### 2.1.4 BODIPY Derivatives

BODIPY dyes are often used as fluorescent trackers for imaging thanks to their high brightness, narrow fluorescence peaks and low sensitivity to changes in pH and polarity. BODIPY structures are highly tunable, and extensions of the π-conjugated system in positions 3- and 5- can lead to strong bathochromic shifts that can be exploited in 2PA. Several commercially available cell stains belong to this class of dyes, such as LysoTracker Red (**55**) which shows 2PA at 1032 nm (Bestvater et al., 2002). BODIPY TR (**56**) is a π-extended derivative with high photostability that was reported with superior 2P brightness (>200 GM) at 1060 nm (Mütze et al., 2012). Extension at the 3- and 5- positions was also the strategy used by Zheng *et al.* to design the compound IR07 (**57)** in 2009 (Zheng et al., 2009). Albeit initially developed for optical power limiting applications, the dye still had a fluorescence quantum yield of 30% and a 2PA cross-section of 101 GM at 1310 nm in CH_2_Cl_2_, which makes it an interesting candidate for further developments in bio-imaging. Interestingly, Prasad and co-workers further extended this aminostyryl-BODIPY with phenylacetylenes, and used compound **58** under 2PE at 1064 nm (Hu et al., 2020). As this dye had a relatively low fluorescence quantum yield of 9% (in THF), the authors investigated its application in 2P photo-acoustic imaging instead of traditional 2P fluorescence.

Theoretical reports have also shown the potential of BODIPY derivatives as 2P and 3P imaging agents (Zhang et al., 2015), which opens the way towards the rational design of NIR-II fluorophores. More advanced dye-design strategies have also been applied to optimise the 2PA properties of BODIPY fluorophores beyond 1000 nm (see Section 2.1.5).

#### 2.1.5 Dipoles, Quadrupoles and Advanced Design Strategies

By taking advantage of strong ICT, relatively simple D-π-A structures can sometimes lead to strongly red-shifted optical properties and important 2PA cross-sections. Importantly, in contrast to symmetrical dyes, their transitions at 2λ_1PA_^max^ are usually allowed, which is an important factor to reach the NIR-II window in 2PA. Triphenylamines are an important ED building block of 2P-responsive dipoles. Examples of triphenylamine dipoles include dye **59** that was incorporated within a poly (styrene-co-maleic anhydride) (PSMA) polymer, thus forming fluorescent nanoparticles (NPs) with high chemical and optical stability across a broad pH range (Alifu et al., 2017). The dipole emits in the NIR-I region, and interestingly shows a bathochromic shift of λ_em_ in the solid state. When excited at 1040 nm (2PA) the PSMA NPs of **59** emit over a wide range of wavelengths (500–950 nm) with a maximum at 678 nm. A large cross-section was measured for these NPs (5.6 × 10^5^ GM), and the fluorescence quantum yield could be tuned from 1.7% to 16.9% by modifying the weight ratio of dye to polymer. These NPs were used as contrast agents to facilitate the 2PA NIR-II imaging of mouse brain blood vessels at the deepest tissue penetration reported to date (Section 3.2, Table 3). Similar to **59**, a second NIR-II absorbing triphenylamine dipole **60** was also utilised for imaging *in vivo* (Section 3.2, Table 3) while acting as a lysosomal photosensitizer for PDT (Zhou et al., 2021). Tuning the EW moiety shifted the 1PA properties of this dipole to the red compared to **59**, allowing 2P imaging to be performed at 1100 nm, i.e. near the 2λ_1PA_ value (530 nm).

Some other amino-substituted D-π-A dyes have been shown to exhibit strong solvochromatic or fluorogenic behaviour (Klymchenko, 2017). A reported water-soluble dipolar fluorophore (**61**) exhibited a large 2PA cross-section value at 1040 nm (440 GM) (Massin et al., 2013). Strong solvato-fluorogenic properties were demonstrated for this dye, with a fluorescence quantum yield of 0.22 in water, and therefore a 2P brightness close to 100 GM. Similar relatively simple structures have led to highly red-shifted 2P active dyes, which constitutes efficient dye design examples. Using a dialkylamino ED group and a pyridinium acceptor in a D-π-A structure led to dipole **62** that was reported with a σ_2_ of 500 GM at 1300 nm (Ricci et al., 2017). Interestingly, the corresponding D-π-A-π-D counterpart **63** was also prepared by the authors. With a C_2v_ symmetry leading to a “bis-dipolar” character, this compound showed an enhanced cross-section at 1300 nm compared to dipole **62**, but the emission quantum yield was reduced by an order of magnitude. With limited quantum yields, both dipole **62** and bis-dipole **63** showed limited 2P brightness. Benzothiadiazole (BTZ) and related derivatives are other typical moieties that have been used in the design of NIR-absorbing chromophores for 1P and 2P bio-imaging, in particular when incorporated as a strongly EW core in a D-π-A-π-D structure. This has led to a strong 2P response with the ability to extend beyond 1000 nm (Yao et al., 2016). However, these pseudo-centrosymmetrical chromophores, with forbidden transitions, can be counterproductive in NIR-II dye design. Moreover, water solubility can be a limitation for these molecules, and they tend to be used more commonly as AIE building blocks (Section 2.1.6), or incorporated in different types of NPs. For this purpose, Liu and co-workers prepared conjugated polymers from BTZ and thiophene derivatives (**64**) that proved highly NIR-emissive once incorporated in phospholipid-type NPs (Wang et al., 2019b). These polymer dots showed significant 2PA between 1000 and 1200 nm, with cross-sections in the 1000–2000 GM range. A 2P brightness of 242 GM was reported, along with impressive *in vivo* results (Section 3.2, Table 3).

Other advanced strategies have been applied to design NIR-II-absorbing 2P dyes with optimised 2PA cross-sections in the NIR-II, without focusing on emissive properties or imaging applications, which has been reviewed recently (Pascal et al., 2021). This includes the preparation of stable π-radical and diradical structures (Hisamune et al., 2015), macrocyclic dyes with controlled topology (Mobius dyes, *meso*-*meso*-linked porphyrin oligomers amongst others) (Tanaka et al., 2008), or multi-chromophoric systems with hybrid electronic transitions (Webster et al., 2009). During their investigation of singlet biradical dyes, Li *et al.* reported the preparation of zethrene derivatives (**65** and **66**) with strong 2PA properties at 1250 nm (Li et al., 2012). The compounds retained fluorescent properties, however, measurements were only performed in chloroform because of their lipophilic nature. Ni *et al.* reported quinodimethane-bridged BODIPY dimers (**67** and **68**) that showed up to 26% diradical character, which provided strong 1PA at the beginning of the NIR-II window at 1100 nm (Ni et al., 2016). The corresponding 2PA band was, therefore, shifted beyond 2000 nm (up to 1500 GM at 2300 nm). Although no application in imaging was envisioned, the authors report that their BODIPY derivatives remain fluorescent in chloroform solutions, with up to a 0.2% quantum yield of infrared emission. This low Φ_f_ value leads to very poor 2P brightness (up to 2.6 GM). The quinodimethane dimerization strategy was also used by Zeng *et al.* to prepare porphyrin dimers with intense 2PA cross-section at 1800 nm (Zeng et al., 2013). In this report, the Zinc and Magnesium porphyrin dimers showed emission bands in the 900 nm range (CH_2_Cl_2_). Self-assembly of porphyrin-oligomers and 4,4′-bipyridine has also led to record cross-sections (up to 2.3 × 10^5^ GM) and strongly red-shifted 2PE (up to 1300 nm) by restricting rotation and enhancing electron transfer through the metal bridge (Drobizhev et al., 2006). Such strategies have led to σ_2_ of several thousands of GM above 1200 nm, but often lead to non-emissive compounds, typically because of preferential vibrational decay or very short excited state lifetimes (Cho et al., 2009). They are also susceptible to poor stability and lack of biocompatibility.

Other families of dyes have also been investigated with the goal of improving 2PA capacity in the NIR-II (Pascal et al., 2021), including for optical imaging applications. The classical dead cell stain propidium iodide (**69**) shows 2PA response at 1015 nm (Bestvater et al., 2002). Polyaromatic fluorophores such as diketopyrrolopyrroles have also been proposed as potential multi-photon imaging agents in theoretical reports (Ye et al., 2017).

#### 2.1.6 Aggregation Induced Emission Dyes and Dots

**FIGURE 4:**
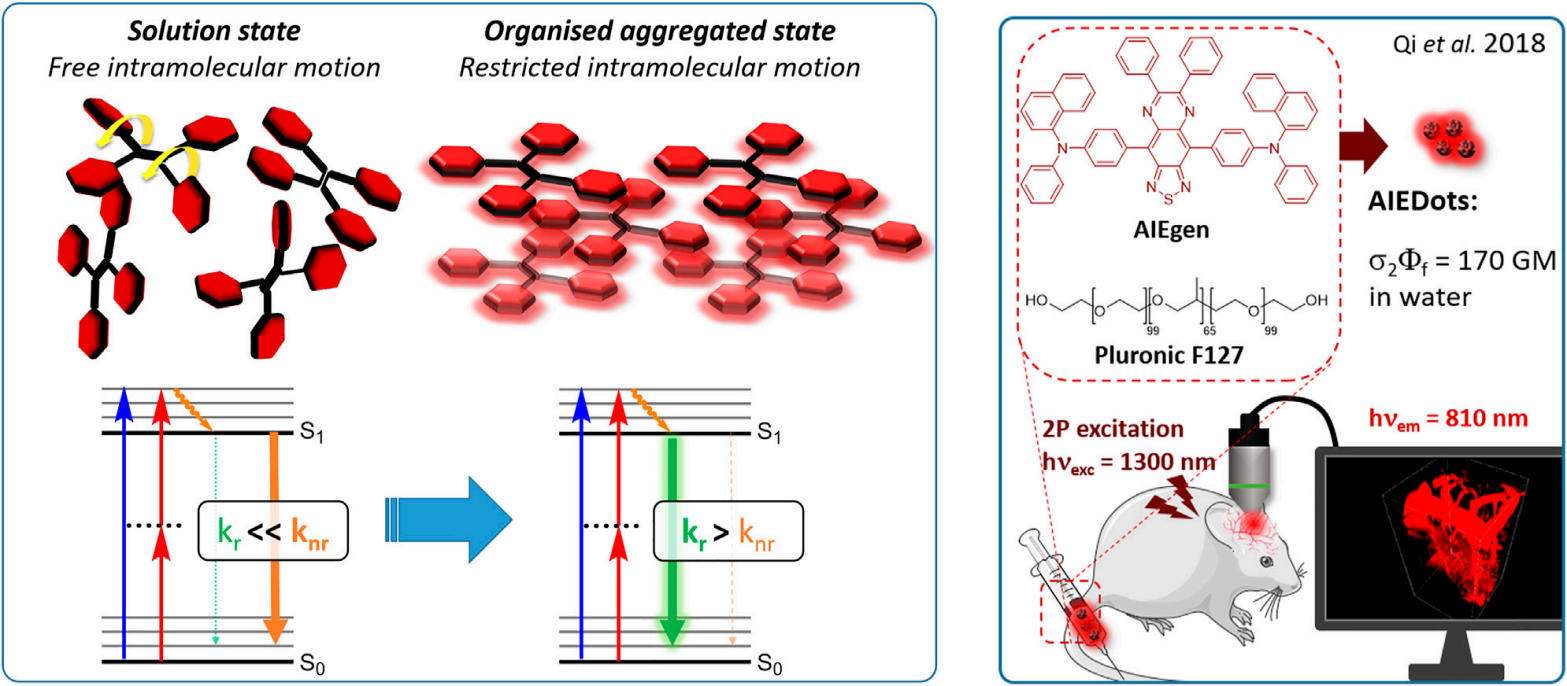
(Left) Illustration of AIEgen molecules in solution and aggregated state, and their corresponding simplified Jablonski diagrams showing the effect of motion restriction on the radiative (k_r_) and non-radiative (k_nr_) relaxation pathways after 1PE or 2PE. (Right) Example of *in vivo* application of 2P microscopy with NIR-II-responsive AIEDots used to reconstruct images of a mouse brain. The 3D reconstruction of mouse brain vasculature is reprinted with permission from ref. (Qi et al., 2018). Copyright 2018 American Chemical Society.

Dye design strategies for 2P-responsive NIR-II fluorophores can lead to highly lipophilic aromatic structures prone to forming irregular aggregates in aqueous environments. Fluorescence quantum yields can thus be dramatically reduced as a result of a reduction in the rate of radiative decay (k_r_), either by significant bathochromic shifts leading to favoured non-radiative vibrational decay (k_nr_) processes, by fluorescence quenching by water molecules, or by intermolecular π–π stacking, which is known as “aggregation-caused quenching”. Counteracting this effect, certain lipophilic organic dyes generate an organised solid-state arrangement of chromophores with a local lipophilic environment within nano-aggregates. While the dye molecules move freely in diluted solution form, the restriction of intramolecular motions (i.e. vibrations, rotations etc.) in this organised aggregated state causes a strong decrease in the probability of non-radiative decay, and therefore a strong increase in fluorescence (Figure 4A, left). The concept of “aggregation-induced emission” (AIE) was first described in 2001 by Tang and co-workers (Luo et al., 2001), and has since become a popular approach to the design of 1P and 2P theranostic agents (Zhu et al., 2018; Yang et al., 2020b; Lu et al., 2020; Han et al., 2021) and circumvent the limitations of standard NIR-dye design strategies. In contrast to traditional organic fluorophores, AIE luminogens (AIEgens) typically exhibit low fluorescence in dilute solutions, but both high Φ_f_ and photostability in the aggregated state, which are key requirements for high-resolution imaging.

In the context of 2P-imaging using NIR-II wavelengths, AIEgens provide a way to prepare highly NIR-shifted chromophores maintaining strong 2P brightness in water. The BTZ derivative **70** has been shown to exhibit AIE behaviour in two different studies, emitting NIR light after 2PE at 1200 nm (aqueous media) and 1300 nm (organic media) respectively (Qi et al., 2018; Liu et al., 2021). Significant cross-section values (1.6 × 10^4^ GM at 1040 nm and 1220 GM at 1300 nm) were reported in the NIR-II for these AIEdots. The emission peak was measured at 810 nm with 14% quantum yield. This AIEgen was used to image mouse brain tissues with high resolution (Figure 4B, right), demonstrating the imaging benefits of NIR-II 2PA over NIR-I 1PA (Section 3.2, Table 3). The use of AIEgen dye **70** was then extended to 2P fluorescent lifetime imaging (2PFLIM) by the same research group (Liu et al., 2021). Replacing the arylamine EDG of this molecule with a propeller-shaped tetraphenylethene moiety (Wang et al., 2019c), a structure commonly used in the design of AIEgens, led to D-π-A-π-D dye **71** that was co-nanoprecipitated with a pegylated phospholipid. The 2PA spectrum of the resulting AIEdots showed a σ_2_ close to 1 × 10^5^ GM both at the maximum of the low energy band (1150 nm) and in the tail of the high-energy band (1000 nm). With a fluorescence quantum yield of 19% in aqueous media and high photo-stability, these AIE dots showed high potential for *in vivo* imaging, which was demonstrated in tumour tissues and blood vessels (Section 3.2 and Table 3).

Outside of the BTZ class of dyes, a 2PA fluorescent nanoprobe made of a triphenylamine-based brominated AIEgen (dye **72**) (Samanta et al., 2021), showing a similar structure to dipole **59** (Section 2.1.5), was reported. The triphenylamine ED group provides a good balance between strong twisted ICT in the molecule and unrestricted rotation to ensure possible AIE. These NPs showed large 2PA cross-section of 3 × 10^3^ GM at 1040 nm and a Φ_f_ of 6%, which also allowed 2D and 3D imaging of brain vasculature (Section 3.2, Table 3). Similarly, four push–pull AIEgens based on a diphenylamine donor attached to different EW moieties (Zheng et al., 2018) were prepared. All 2PA bands extended somewhat past 1000 nm, but the highest cross-sections were obtained with the isophorone and furanone dipoles **73** and **74** (887 GM at 1020 nm for the latter) which showed great promise in *ex vivo* cell and tissue studies. Finally, the tetraphenylethene propeller-shaped moiety was attached to a BODIPY dye to prepare AIEgen **75**. The NPs prepared from this dye showed a record absorption capacity past 1000 nm (2.9 × 10^6^ GM at 1040 nm) (Wang et al., 2015), which is among the highest cross-section values in the NIR-II region all classes of materials combined. Therefore, AIEgens allow the combination of massive cross-sections, arising from the combined cross-section contribution of each dye in the NP, and of a fluorescence strongly switched-on in water. Both these factors combine to give unprecedented 2P brightness values (>10^4^ GM), which makes them high-potential materials for 2P imaging in the NIR-II (Section 3.2, Table 3).

### 2.2 Carbon, Hybrid and Inorganic Nanomaterials

Carbon and metal-containing nanomaterials have been investigated for decades as a source of 2P-reponsive imaging agents thanks to unique electronic, physical and morphological properties. Such materials also tend to show higher brightness and photo-stability than organic dyes, and thanks to their surface functionalisation, they provide huge versatility to expand into multimodal and theranostic applications. The potential toxicity of metal and carbon nanomaterials is arguably the main limitation to their use for *in vivo* imaging.

Carbon nanomaterials have been a key focus of research in bio-imaging over the past decade owing to their unique optical properties, large surface area, and robust photostability allowing long-term imaging. Carbon dots (CDs), single-wall carbon nanotubes (SWCNTs), graphene derivatives and nanodiamonds have all been reported as luminescent 2P-active materials with imaging potential (Hong et al., 2015). In the NIR-II window, semiconducting SWCNTs have demonstrated strong potential, both because of their strong 2P response, and their NIR emission. In the context of optoneurology and neurotransmitter sensing, a dopamine-sensitive nanosensor was developed using SWCNT (**76**), and provided chirality-dependent fluorescent turn-on responses varying between 20% and 350% in the presence of the analyte (Bonis-O'Donnell et al., 2017). Sensing was performed at 1560 nm under 2PE with a σ_2_ estimated at 2.16 × 10^5^ GM, but the fluorescence quantum yield of **76** remained limited (Φ_f_ = 0.0023). These nanosensors were embedded 2 mm into strongly scattering tissue phantoms mimicking brain tissues, which demonstrated that the light scattering decreased from 42% to 4% using NIR-II 2PE compared to traditional 1PE. In 2014 an aptamer-modified graphene oxide material (**77**) was used to show excitation dependant luminescence outputs and develop multi-channel and multi-colour imaging of multi-drug resistant bacteria (Pramanik et al., 2014). In particular, 2PE of the material at 1120 nm led to bright red-light emission, which allowed imaging of methicilin-resistant *Staphylococcus Aureus* (MRSA) with a σ_2_ above 3.6 × 10^4^ GM in aqueous solution. Recently, nitrogen-doped graphene quantum dots were also reported with high luminescence and photosensitizing properties for antimicrobial applications (Kuo et al., 2022). Although the 2P properties of these graphene-based nano-objects were only measured up to 970 nm, they likely still possess 2P responses beyond 1000 nm. Finally, sulfoxide- and carbonyl-enriched CDs (**78**) were prepared by solvothermal treatment of readily available citric acid and urea. These CDs were brightly fluorescent at 760 nm under 1PE at 714 nm, with efficient NIR-II absorption upon 2P and three-photon (3P) excitation at 1200 and 1400 nm (Li et al., 2018). Carbon quantum dots prepared from tris(4-aminophenyl)amine (**79**) also led to ultra-narrow emission at 615 nm with high photoluminescence quantum yield (84%) (Liu et al., 2020). These CDs were used for *in vitro* for 2P imaging of tumor spheroids at 1100 nm, with a penetration depth reaching 200 μm. Further 2P *in vivo* imaging was carried out in zebrafish larvae, in which a maximum penetration depth of 500 μm was achieved (Section 3.2, Table 3).

Noble metal nano-objects are known to interact strongly with high-intensity light pulses, which is the source of interesting NLO properties (Olesiak-Banska et al., 2019). In recent reports, gold nanoparticles (AuNPs) have been used to develop a hybrid theranostic platform (**80**) combining anti-GD2 antibodies and SWCNTs for selective 2P imaging and efficient photothermal therapy of human melanoma cancer cells at 1100 nm (Tchounwou et al., 2015). Although no σ_2_ or Φ_f_ values were explicitly mentioned in the article, the authors measured the 2P-induced photoluminescence of the hybrid nanomaterial and evidenced that the strong plasmon-coupling generated by the gold increased the emission by 6 orders of magnitudes compared to the AuNPs or SWCNTs alone. The plasmon coupling also acted as a local nano-antenna to enhance the photothermal efficiency of this theranostic system. Recently, hybrid Au-Si NPs functionalised with a NIR-resonant cyanine dye were also exploited for tissue imaging with surface-enhanced resonance hyper-Raman scattering (SERHRS) (Olson et al., 2022), a vibrational 2P spectroscopy technique.

Gold nano-clusters (AuNCs) are ultrasmall nano-materials constituted of as little as a dozen atoms of gold. Due to the proximity of their size to the de Broglie wavelength of an electron, they do not exhibit the typical plasmon resonance observed in larger AuNPs, but instead show strong quantum confinement effects, leading to molecule-like electronic transitions and fluorescence properties. AuNCs with a number of gold atoms varying from 25 to 2406 had their 2P fluorescence properties measured in the NIR-I and -II regions (Ramakrishna et al., 2008). In the NIR-I (800 nm), 2PA cross-sections ranging from 5 × 10^5^ to a 3 × 10^6^ GM were reported; but interestingly, the σ_2_ value per gold atom decreased drastically with the size of the AuNC, eventually showing saturation when transitioning to standard AuNP behaviour. In the NIR-II, the Au25 clusters (**81**) showed a cross-section of 2700 GM at 1290 nm with emission of light at 830 nm, however the quantum yield value of such systems is said to be in the 10^–7^ range which limits their brightness. PEG-lipoic acid functionalised AuNCs (**82**) of 1.5 nm in size have also been discussed for cellular imaging (Oh et al., 2013). The 2PA cross-section of these AuNCs was above 300 GM at 1100 nm, and remained >100 GM at 1300 nm where the measurement ended. Fluorescence at 820 nm with a Φ_f_ of 4–8% was reported depending on the surface functional group, and the objects were stable for months and generally non-toxic.

**FIGURE 5:**
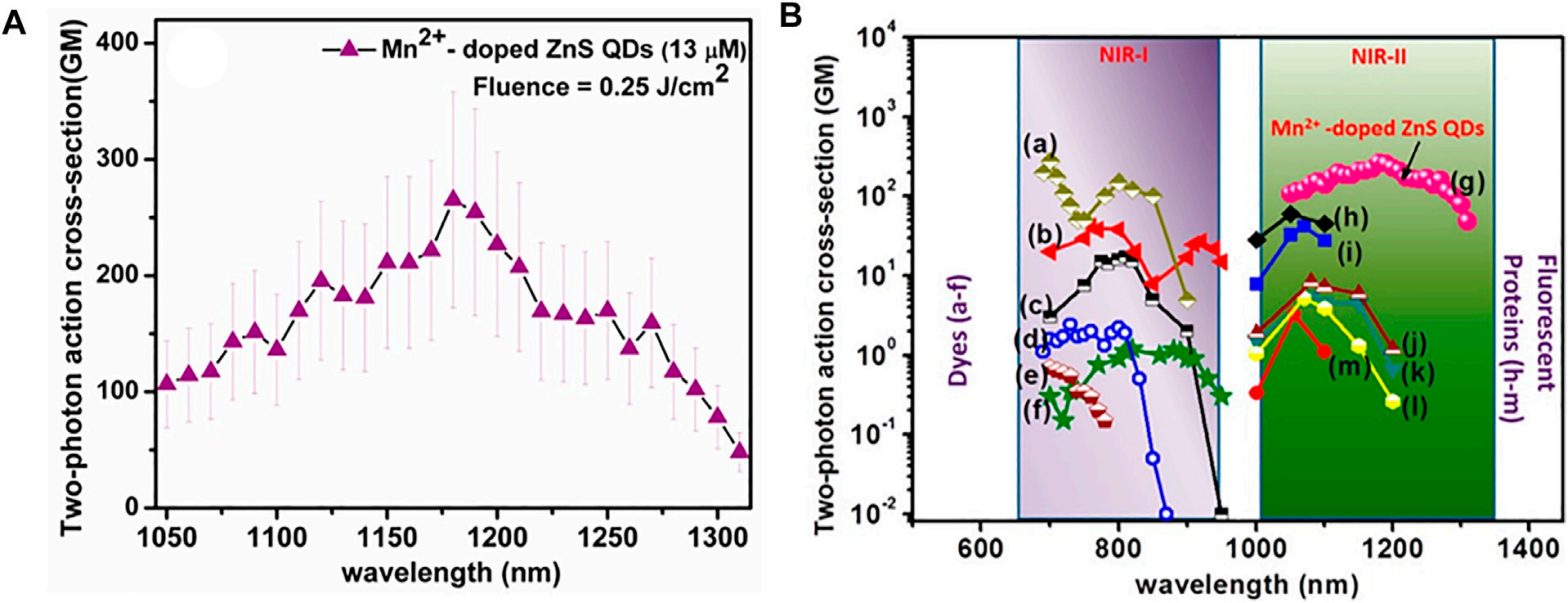
**(A)** 2PA spectrum of Mn^2+^-doped ZnS QDs in the range from 1050 to 1300 nm reported by Subha *et al.* **(B)** Comparison of σ_2_ values in Mn^2+^-doped ZnS QDs (curve g) with other standard chromophores (curves a–f) and fluorescent proteins (curves h–m): (a) Rhodamine B, (b) Fluorescein, (c) Coumarin 307, (d) Cascade blue, (e) Dansyl and (f) Lucifer Yellow), and (h) tdTomato, (i) mBanana (j) mRFP (k) mCherry (l) mStrawberry (m) mTangerine). Reprinted with permission from ref. (Subha et al., 2013). Copyright 2013 American Chemical Society.

Quantum dots (QDs) are crystalline semiconductor materials that also display quantum confinement effects due to their nanometer size (Wegner and Hildebrandt, 2015; Barroso, 2011). The dependency of the confinement energy on the QD’s diameter leads to size-dependent absorption and emission, with smaller NPs resulting in larger band gaps–and therefore blue-shifted emission, and larger NPs having more red-shifted emission. Their highly tunable size and properties, high stability, limited photobleaching, and reported 2PA cross-sections (>5.0 × 10^4^ GM at ≥1000 nm) make them great candidates for OMI applications (Gui et al., 2017; Nyk et al., 2012). Due to their unique semiconducting energy profile, they also feature broad absorption bands which would make them ideal for 2PA NIR-II imaging where excitation wavelength can be laser-dependent (Section 3.1). The main drawbacks of QDs arise due to reports on potential toxicity related to their heavy metal components (Tsoi et al., 2013). Examples of 2P-responsive QDs in the NIR-II include Mn^2+^-doped ZnS QDs (**83**) reported in 2013 by Subha *et al.* (Subha et al., 2013), which presented 2P- and 3P-induced photoluminescence at 586 nm resulting from the electronic transitions of the manganese ions. A maximum 2PA cross-section of 265 GM was measured at 1180 nm, with an absorption band extending beyond 1250 nm (Figure 5A), which was higher than most standard dyes and fluorescent proteins (Section 2.3) reported at the time (Figure 5B). These QDs also possessed long photoluminescence lifetime (millisecond range).

Pioneering work combining 2P fluorescence and 2PFLIM using PbS/CdS QDs (**84**) as water-dispersible contrast agents was recently reported (Ni et al., 2022). These QDs allowed “NIR-to-NIR” imaging under 2PE at 1550 nm with a σ_2_ of 530 GM in water. Importantly, the QDs maintained bright fluorescence properties at 1270 nm, with a quantum yield of 18% in water, which is higher than most organic dyes at such long wavelengths. The high fluorescence lifetime (τ = 501 ns) of these QDs allowed the authors to implement a 2PFLIM system to complement 2P fluorescence imaging, and *in vivo* images of mouse brain blood vessels were obtained for the first time. As well as this, it is worth mentioning that Larson *et al.* reported polymer-encapsulated CdSe-ZnS QDs with 2P brightness of up to 4.7 × 10^4^ GM, which is orders of magnitude higher than organic fluorescent probes (Larson et al., 2003). Although the measurements were only performed between 700 and 1000 nm at the time, brightness values proved relatively constant all over this range of wavelength thanks to their large absorption band; and QD605 (**85**), the brightest NP in their study, would likely still respond in the NIR-II regime.

### 2.3 Fluorescent Proteins

**FIGURE 6:**
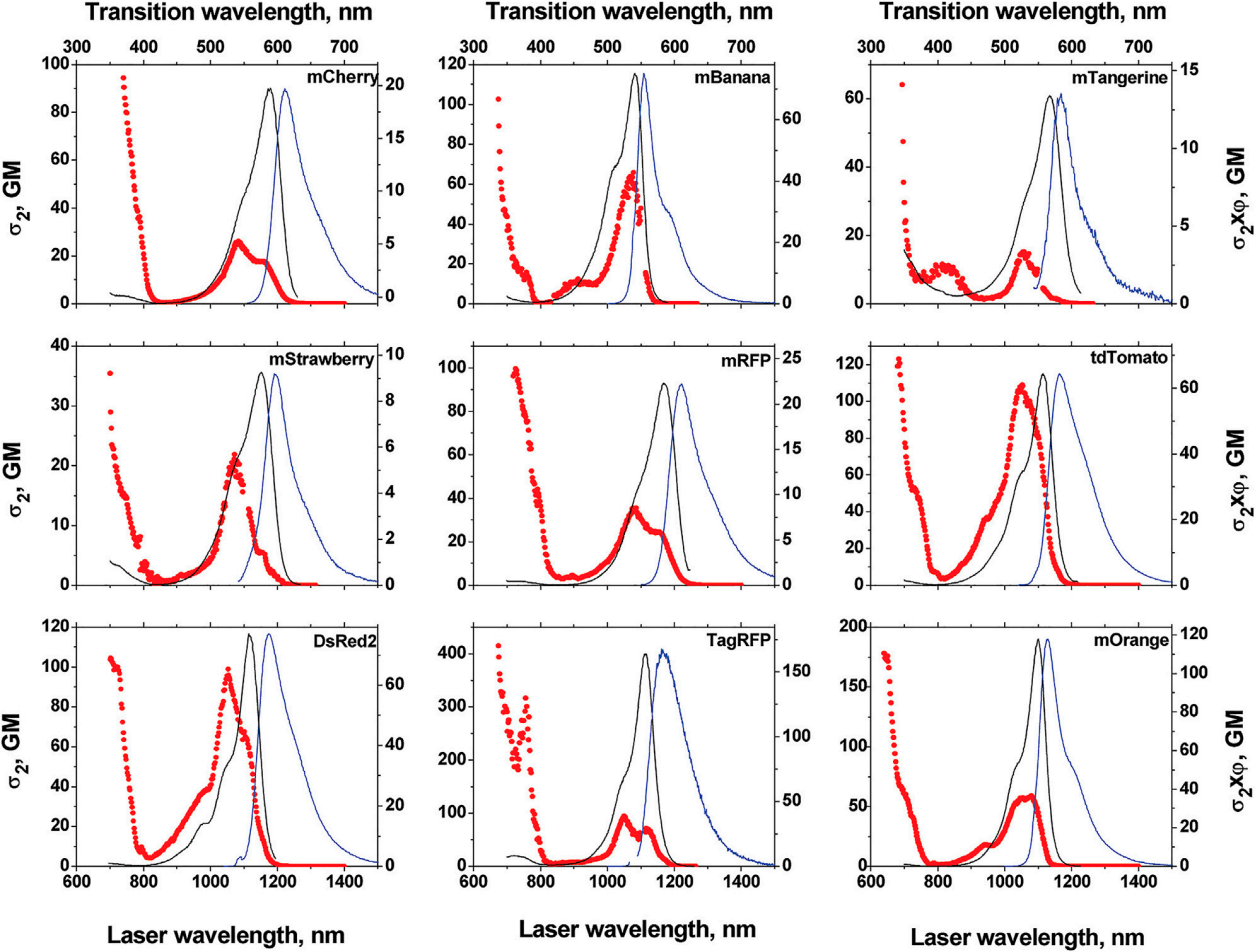
1PA and 2PA spectra of fluorescent proteins of the “fruit series”. Left axis shows the 2PA cross-section σ_2_^(λ)^ per mature chromophore, right axis shows corresponding σ_2_^(λ)^Φ_f_ values. Adapted with permission from ref. (Drobizhev et al., 2009). Copyright 2009 American Chemical Society.

FPs are a class of proteins that contain chromophores that arise from specific amino-acid sequences in their polypeptide sequence undergoing a series of atypical transformations (Tsien, 1998; Shaner et al., 2004). Unlike the aforementioned examples that require injection or incubation with a contrast agent, FPs can be genetically encoded, and they can therefore generate luminescence with high target specificity. The 2P properties of NIR-II-responsive FPs have been well documented in the seminal work by Drobizhev et al. (2011). Several proteins absorbed efficiently above 1000 nm, and we limited our selection to the ones showing brightness values above 5 GM in Table 1 (compounds **86–104**). Among them, the so-called “fruit proteins” have been reported with medium to strong 2P brightness in the 1000–1200 nm region (Figure 6) (Drobizhev et al., 2011; Drobizhev et al., 2009). With a maximum 2PA cross-section of 278 GM at 1050 nm, as well as an absorption band extending beyond 1200 nm, tdTomato (**86**) was reported as the brightest in the series (Shaner et al., 2004; Drobizhev et al., 2009). Proteins tdKatushka2 (**87**) and dsRed2 (**88**) follow with brightness values in the 50–100 GM range. As shown in Figure 6, the σ_2_Φ_f_ values per mature chromophore of FPs are comparable to those of organic fluorophores, and orders of magnitude lower than those of nanomaterials and AIEgens. Still, as highlighted by Drobizhev et al. (2011), these 2PA properties match well with current widespread NIR-II laser technologies such as Ti:Sapphire with optical parametric oscillators, neodymium (Nd)- and ytterbium (Yb)-doped fibre and glass lasers and chromium-forsterite (Cr-Mg_2_SiO_4_) lasers (Section 3.1) (Shaner et al., 2004; Drobizhev et al., 2009). FPs were therefore among the first luminescent trackers to be used successfully to perform 2P imaging of biological systems in the NIR-II window, and to develop new adapted multi-photon technologies. Gene expression in transgenic zebrafish embryo, tagged with a red FP (Hc-RFP, **89**), was investigated by Tsai *et al.* using 2P imaging (Tsai et al., 2005; Tsai et al., 2006). Heart-specific regulatory elements of a zebrafish cardiac gene fused with the encoding element for Hc-RFP were injected into zebrafish embryos. This generated a zebrafish line that showed strong 2P red fluorescence in cardiac cells (Tsai et al., 2005). This red FP allowed imaging with a 1230 nm fs light source, providing superior imaging resolution compared with traditional green FP-based 2P microscopy. In addition, the properties of HcRFP were measured under 2PE by the same group, showing an excitation maximum around 1200 nm and an absorption cross-section of the same order as the green FP (GFP) (Tsai et al., 2006). The use of red FPs and mCherry (**90**) under OPO excitation has also been demonstrated (Herz et al., 2010) and compared against NIR-I activated GFP. The authors report that, in the cortex of fluorescent-protein-expressing mice, a maximal imaging depth of 508 μm was possible when imaging with tandem dimer RFP (TdRFP, **104**) at 1110 nm, which represents an 80% enhancement compared to GFP-expressing mice imaged at 850 nm. The depth-dependent deterioration of the spatial resolution was also significantly lower at 1110 nm. Yang *et al.* induced the expression of the tdTomato protein in excitatory neurons of mice (Yang et al., 2013). The authors used this model to validate the development of their multi-colour OPO-based laser set up which was used successfully at 1070 nm for 2P imaging of the intact brain cortex (Section 3). Voigt *et al.* recently validated the design of a novel semiconductor-disk laser (SDL) by performing *in vivo* 2P imaging of FP-containing *Drosophila* larvae (Voigt et al., 2017).

## 3 *In vivo* 2P-NIR-II Bio-Imaging: Instrumentation and Examples

Two-photon absorption is a third-order NLO phenomenon. As such, 2PA can be observed in many types of materials provided that the intensity of the electric field in the light source is strong enough to generate a non-linear polarisation in the material (He et al., 2008).

For 2PA imaging applications, high photon densities are required to ensure sufficient fluorophore excitation; requiring around one million times more photon flux compared to 1PA events (Le Bot, 2009). This involves the use of high intensity pulsed (mode-locked) lasers. Such systems provide brief but intense light pulses which results in high photon fluxes arriving at a sample to promote 2PE, while also keeping the average power low enough to prevent tissue damage. By reducing the average excitation power, the number of 1PA events are minimised, which is known to be the source of heating and resultant photo-damage. Laser requirements for 2P imaging are typically met with output powers of >1 W, pulse durations of <100 fs, repetition rates of typically between 80-100 MHz, and high peak powers (>100 kW). Lasers capable of producing pulses of light of durations shorter than picoseconds (10^–12^) do so using a method known as ‘mode locking’.

Recent years have shown an increase in successful 2PA NIR-II imaging with imaging depths of up to 1200 μm into tissue being claimed (Alifu et al., 2017). However, most lasers explored in current literature have their output wavelengths in the NIR-IIa region, with most reports showing imaging performed at lasing wavelengths of <1300 nm (Section 3.2, Table 3). This is likely due to the limited capacities of current lasers struggling to reach the power, portability and flexibility requirements at such long wavelengths.

Traditional systems for 2P imaging involve the use of solid-state lasers (Drobizhev et al., 2011; Ustione and Piston, 2011). However, due to their drawbacks, such lasers have limited applications in the NIR-II window. As such, lasing systems involving the use of optical parametric oscillators (OPOs), optical parametric amplifiers (OPAs), and fibre lasers, are more commonly used for 2P NIR-II imaging.

### 3.1 NIR-II Laser Technologies

#### 3.1.1 NIR-II Solid-State Pulsed Lasers

The first paper demonstrating 2P imaging applications was demonstrated by Denk et al. (1990) in which a 25 mW colliding pulse, mode-locked dye laser with an emission of 630 nm was used. Historically, dye lasers dominated the field of tuneable lasers but were then replaced by solid-state lasers (often based on Ti:Sapphire crystal) due to their bulky structures, complex liquid handling systems, and the involvement of toxic and volatile dyes (Ferguson et al., 1993).

Solid-state ‘mode-locked’ lasers compatible with 2P imaging produce high-power light pulses on a femtosecond time-scale. Solid-state lasers typically consist of a solid gain media typically doped with rare-earth ions, such as ytterbium, chromium, and neodymium, and optically pumped by a diode laser (Chénais and Forget, 2012; Arbuzov et al., 2013). Solid-state ‘mode-locked’ lasers have been historically used in imaging applications for their convenience, high potential output powers, and low cost. However, their applications in the NIR-II is restricted due to their shorter emission wavelengths or limited wavelength tunability (Zhang et al., 2021b). Ti:Sapphire lasers have a range of advantages such as a wide tuning range, high output power, and femtosecond pulsing capability, however, they are not suited for 2P NIR-II imaging applications due to their emission wavelength (700–1000 nm) not reaching the NIR-II optical window.

Solid-state lasers based on crystals such as Cr:Forsterite are of particular interest as they can produce emission wavelengths between 1220 nm and 1270 nm and have been successfully applied in 2P NIR-II imaging applications with wavelengths of around 1230 nm (Shen et al., 2015; Tsai et al., 2005). Nd:YLF lasers are a common composition that produces a fixed lasing wavelength of 1047 nm (Squirrell et al., 1999; Wokosin et al., 1996b). As demonstrated in Section 2, the 2PE wavelength at 1047 nm overlaps with the absorption band of several bio-imaging dyes. However, both Cr:Forsterite and Nd:YLF mode-locked lasers lack a wide wavelength tuning range (Trägårdh et al., 2016), which implies that fluorescent probes must be carefully chosen in order to match the small bandwidths, therefore this tends to limit biological imaging to just one colour. An example of a solid-state laser being used for *in vivo* applications involve a Nd:YLF laser being used to perform 2P NIR-II imaging of a stained zebrafish embryo (Wokosin et al., 1996a) (Section 3.2, Table 3). To overcome the drawback of limited excitation wavelengths of such solid-state lasers, multi-colour 2P imaging can instead be achieved by using two tunable lasers in tandem, although this is a relatively high-cost solution. Other solutions have been found in utilising the second Stokes shift to extend output wavelengths in Ti:Sapphire laser systems, or in methods such as phase-shaping (Brenner et al., 2013; Trägårdh et al., 2016).

#### 3.1.2 NIR-II OPO/OPA Systems

OPOs based on the second-order NLO interactions can convert the output wavelength of an input laser (pump) into two longer wavelengths with lower energy output waves, known as signal and idler (Trovatello et al., 2021). Both continuous wave (CW) and pulsed OPOs can be realised depending on requirements and applications (Sowade et al., 2013). In phase-matching conditions, parametric amplification occurs within a NLO crystal where all three waves are interacting (Trovatello et al., 2021). Wavelengths of the amplified signal can be tuned to access the near-, mid-, and far-infrared regions–which is often much harder to achieve in traditional laser systems. OPO systems have great potential in biological imaging applications as a wide range of wavelengths from a single laser allows multicolour imaging across the whole NIR spectrum, overcoming the limitations of standard solid-state systems. Such systems have only been commercially available within the last few decades, and have now extended lasing applications into the deep IR (up to 2500 nm) (Pascal et al., 2021), which has enabled the discovery of new 2P-absorbing organic molecules within this spectral range where other associated tools for optical investigation exist.

Traditional ‘mode-locked’ lasers in combination with OPO techniques now appear to be the laser system of choice for 2P NIR-II imaging applications (Table 3). Common 2P microscopes include a high-peak-power Ti:Sapphire laser used in combination with a compatible OPO, which can significantly extend the wavelength range. For example, the Blaze laser from Radiantis offers a “one box” Ti:Sapphire/OPO system with three output ports (pump, signal and idler). The pump laser (Ti:Sapphire) provides a tunable wavelength range of 730–1020 nm, the signal output provides a tunable wavelength range of 1000–1550 nm, and the idler output provides a tunable range of 1620–4000 nm. Unfortunately, the output power of the tunable OPO laser decreases dramatically when tuned to longer wavelengths due to the lower conversion efficiency and higher intrinsic loss. Here, the power drops from 2.2 W to 250 mW with increasing wavelength, thus limiting its imaging applications. Due to the difficulties in developing long-wavelength lasers that also match the power requirements for 2P imaging, most current examples in the NIR-II window are demonstrated with wavelengths between 1000-1300 nm. An example of the benefits of OPOs was demonstrated by Herz *et al.* who showed the increased tissue penetration performance by using an OPO laser compared to a traditional Ti:Sapphire laser (Herz et al., 2010). Recent advancement has led to the commercialisation of systems capable of 2P NIR-II imaging with the integration of OPO based lasers. The Olympus FVMPE-RS Multiphoton Microscope appears to be a popular system of choice for current 2P NIR-II *in vivo* imaging purposes, achieving impressive resolution and penetration depths (Section 3.2, Table 3) (Zhou et al., 2021; Wang et al., 2019b; Wang et al., 2019c; Liu et al., 2020). This microscope can image at wavelengths of up to 1300 nm and also contains detectors and other equipment required for plug-in bench top imaging. This is achieved by combining two lasers within the microscope, namely the MAi Tai DeepSee One Box ultra-fast laser (Ti:Sapphire-based for excitation up to 1040 nm), and an InSight® DS+™ OPO (up to 1348 nm). Also worth noting is the Leica TCS SP8 DIVE upright multiphoton/confocal microscope that claims to be the first multiphoton microscope with spectrally tunable detection. This microscope utilises an OPO laser source to realise a tunable output wavelength between 680-1300 nm and has also demonstrated 2P NIR-II imaging applications *in vivo* (Section 3.2, Table 3) (Liu et al., 2020).

Limitations of OPO lasers arise due to the requirements of a pump source with high spatial coherence and optical intensity, often requiring a diode-pumped solid-state laser. Furthermore, complex procedures have to be undertaken that require variation in the crystal’s temperature, orientation, and poling period in order to realise phase matching. Careful free-space alignment and temporal synchronisation are also required for the OPO cavity, which makes the system sensitive to external perturbation and hence a high-level of maintenance is needed. Power restraints, requirements of water cooling, bulkiness, high cost, and expensive maintenance requirements are further limitations for desirable “turn-key” 2P microscopy. To overcome these downfalls, other systems have been developed, such as OPAs. Although OPA systems have similar principles of converting the short-wavelength input pump to the output signal with wavelength in the NIR-II regime, they do not need cavity and temporal synchronisation which can result in a simpler structure and has a smaller footprint (Chen et al., 2020).

Yang *et al.* demonstrated 2P NIR-II imaging by using a multi-colour ultrafast OPO source (Yang et al., 2013), where brain tissues of a tdTomato-expressing mouse were imaged with 2PE at 1070 nm (Section 3.2, Table 3). Xu *et al.* have developed a periodically poled lithium niobate OPA system operating at 2-ps pulse duration (Xu et al., 2021). In their work, imaging at a depth of 40 μm was achieved in both label-free coherent anti-Stokes Raman scattering (CARS) and 2PE based imaging of mitochondrial flavin adenine dinucleotide autofluorescence in tissue samples.

#### 3.1.3 Fibre Lasers

Fibre lasers were first demonstrated in the mid-1980s, followed by the development of high energy Q-switched fibre lasers and mode-locked fibre lasers (Rose, 2019). Fibre lasers consist of an optical fibre doped with rare-earth ions such as Erbium, Neodymium, or Ytterbium, similar to the elements used in their solid-state counterparts (Kim et al., 2012; Mohr et al., 2020). Light from a pump source is guided through this robust waveguide that provides a long gain medium length, resulting in a high optical gain (Rose, 2019).

**FIGURE 7:**
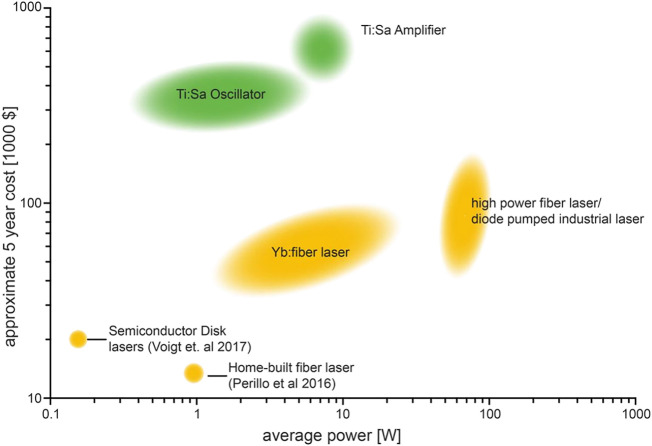
The approximate 5-years costs of traditional tunable 2PA lasers (green) and alternative fixed wavelengths lasers (yellow), demonstrating the economical benefits of fixed wavelength fibre lasers. Reproduced from the supplementary material of ref (Mohr et al., 2020) with permission from the authors.

Fibre lasers present unique optical and practical benefits for 2P NIR-II imaging by removing the requirement of bulk optics and free-space alignment, which offers the benefits of compactness, high stability, reduced initial and maintenance cost (Figure 7), and increased reliability compared to OPO lasers (Table 2). Simple air-cooling of fibre lasers is possible, due to the large surface-area of the fibre, compared to expensive and cumbersome water-cooling that traditional solid-state lasers require. This all results in greater flexibility for the end-user, with many in the near-future looking likely to replace the bulky and complex laser systems and provide a more ‘plug and play’ approach to 2P microscopy imaging.

Importantly, unlike solid-state lasers and OPO lasers that tend to decrease output power at longer wavelengths, fibre lasers can maintain high output powers at the wavelengths required for 2P imaging. This was demonstrated by Jung *et al.*, whose initial attempts of the 2PE of Chrimson-expressing neurons with a conventional femtosecond Ti:Sapphire laser did not result in reliable activation of the target neurons (Jung et al., 2020). On the contrary, activation was observed upon 2P NIR-II excitation at 1070 nm (2W) by using a Yb-doped fibre laser which provided 5–10 times higher power output than the Ti:Sapphire laser. Another example of 2P NIR-II imaging was demonstrated by Kim *et al.*, showing tridimensional 2P imaging of *ex vivo* nerve cells at 1060 nm using a Yb-doped fiber lasers (Kim et al., 2012).

The major challenge of developing high-power short-pulsed fibre lasers is to prevent the distortion of pulse shapes by nonlinear effects such as self-phase modulation and stimulated Raman scattering, which can consequently degrade peak power and 2PE efficiency (Liu and Yang, 2012). This could reduce the fluorescence signal and cause unwanted heating within the sample, and as such, certain technical approaches such as temporally stretching the pulse (Eisele, 2020) can be implemented to resolve this limitation. The tuning range of common fibre lasers for 2P imaging is below 100 nm due to the limited gain bandwidth. Current commercially available fibre lasers using common doping agents only provide distinct wavelengths of around 780 nm, 920 nm, 1050 nm, and 1550 nm (Eisele, 2020). Commonly used Yb-doped fibre lasers for 2P NIR-II imaging have a tunability range of around 1020–1080 nm (Liu and Yang, 2012). However, although difficult, methods for expanding the tunability range of fibre lasers are being explored by implementing nonlinear effects as well as temperature and magnetic field control (Wei et al., 2021). Zhang *et al.* also recently developed a CW fibre laser with an output wavelength tunable from 1000 to 1900 nm by utilising a random distributed feedback Raman fibre laser.

So far only a limited number of biological 2P NIR-II studies have been reported with fibre lasers despite their portability, ease-of-use, access to longer wavelengths, and high power features, possibly due to the lack of commercially available microscopes that integrates such lasers. However, *in vivo* studies using in-house built fibre lasers have demonstrated their high potential, both by performing 2P NIR-II imaging at 1550 nm–the longest wavelength so far reported (Berezin et al., 2011; Ni et al., 2022), and by reaching the deepest *in vivo* penetration (1040 nm) (Alifu et al., 2017) (Section 3.2, Table 3). In addition, the components of fibre lasers are much cheaper than OPO sources, as evidenced by Perillo *et al.* who developed a 2P NIR-II microscope using a mode-locked Yb-doped fibre laser for ∼$13000 and applied it to image FPs at 1060 nm with penetration depths of up to 900 μm (Perillo et al., 2016). In contrast, the cost of a OPO-based imaging system commonly used for similar 2P NIR-II applications, such as the Olympus FVMPE-RS (Section 3.2, Table 3) can be up to 10–100 times higher. Modern solutions will hopefully arise in time with the development of affordable commercial benchtop fibre lasers suitable for 2P NIR-II clinical applications.

### 3.2 *In vivo* Examples of 2P NIR-II Imaging

Clinically relevant examples of 2P NIR-II imaging have been reported using commercially available biological stains, FPs, and custom-made AIEgens. This section aims to discuss the probes presented in Section 2 in a photo-biological context, with a focus on *in vivo* examples.

The main aim of 2P NIR-II imaging is to improve resolution and depth perception when imaging. The latest examples of 2P NIR-II emitting materials being used *in vivo* (“*in vivo”* here indicating agents being administered in a living creature; including examples of imaging post-resection) can be seen in Table 3. As well as having outstanding applications for bio-imaging, recent advances in NIR-II 2PE have led to developments in other biological applications such as PDT and photoacoustic imaging (Wang et al., 2020b; Hu et al., 2020; Ma et al., 2021b).

In 1996, Wokosin *et al.* demonstrated the improved 2P NIR-II imaging resolution at a penetration depth of 55 μm into stained zebrafish embryos compared to traditional 1P confocal microscopy (Wokosin et al., 1996d). In this case, a traditional solid-state Nd:YLF mode-locked laser operating at 1057 nm was used to image safranin-stained embryos (a biological stain typically used for histology and cytology with an absorption and emission of ∼500 nm and ∼590 nm respectively) (Krumschnabel et al., 2014). Zebrafish have a history of being used for biological imaging due to their transparency resulting in limited photon scattering. In the more recent work of Liu *et al.*, a penetration depth of 500 μm was reached in zebrafish larvae using red-emitting CDs **79** excited at 1100 nm using a commercial microscope (Leica SP8 DIVE) fitted with an OPO system for 2PE (Liu et al., 2020). DTTC (**30**) is another example of a commercial stain used for 2P NIR-II imaging, where a fibre laser was used to achieve an imaging depth of up to 210 μm at 1550 nm in a resected mouse kidney post-injection (Berezin et al., 2011).

**FIGURE 8:**
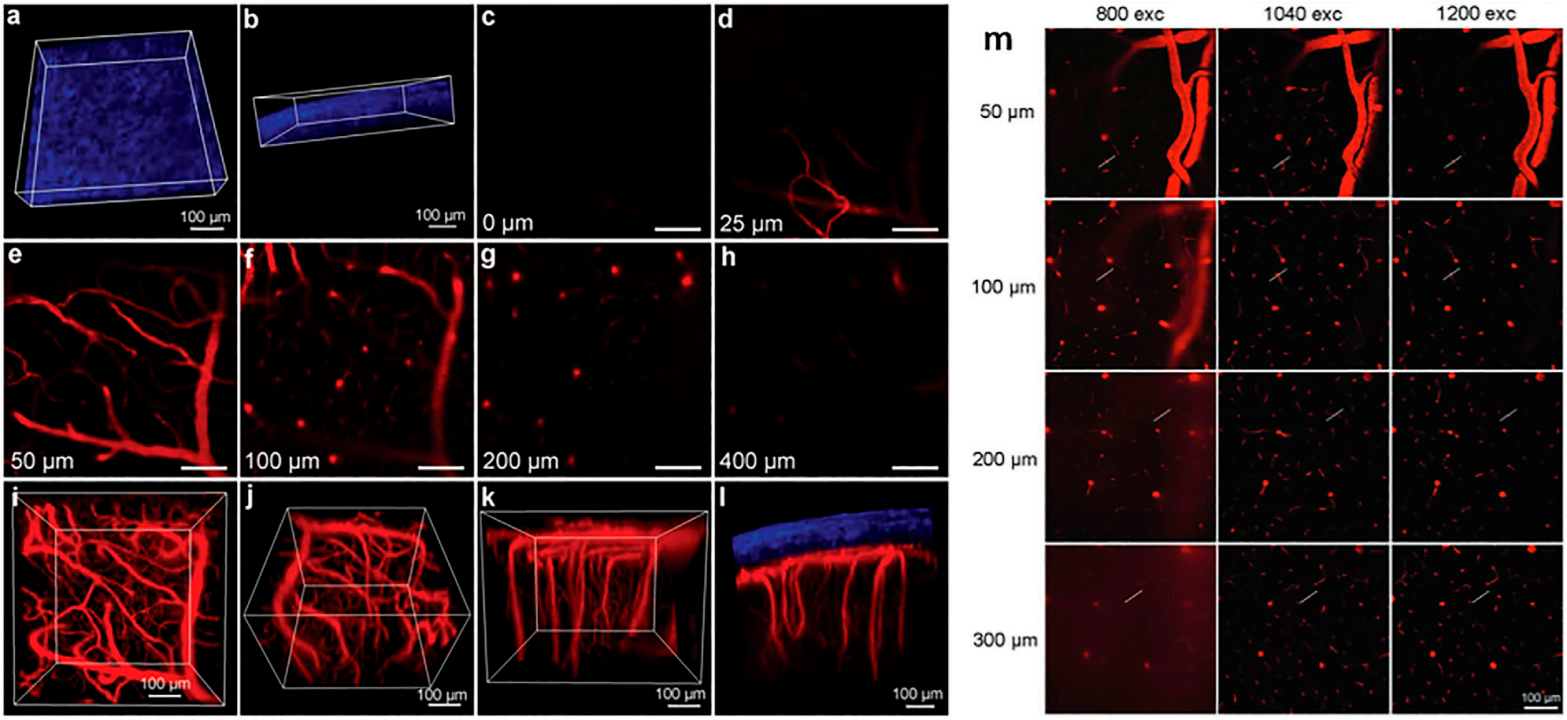
2PA images of brain blood vessels in a mouse injected with CP dots. **(a,b)** 3D reconstructed second-harmonic generation images of mouse skull. **(c–h)** 2PA images at various vertical depths (0–400 μm) at 1200 nm excitation, 660–750 nm emission. **(i–l)** 3D reconstructed 2PA images of brain blood vessels. **(m)** comparison of 2PA images at excitation wavelengths of 800, 1040, and 1200 nm, of brain vasculature in a mouse injected with polymer dots. 2PA line intensity profiles with the different wavelengths across the blood vessels on the right were also acquired for the corresponding depths. Emission at 660–750 nm. Reprinted from ref (Wang et al., 2019b). with permission from John Wiley and Sons.

Out of all *in vivo* studies reported in this range of wavelengths, AIEgen NPs and other NP-based systems appear to be the most popular choice to demonstrate superior penetration depth compared to other systems (Table 3). In the context of *in vivo* 2P imaging, NPs bypass the solubility limitation of organic dyes. They also show increased stability and cumulative size-related increase in 2PA cross-section; all of which make them promising imaging agents. The current record of tissue penetration was thus reported by Alifu *et al.* (Nowak-Król et al., 2013) using triphenylamine dipole **59** incorporated within PSMA-based NPs, which led to 2P imaging at a depth of 1200 µm into mouse brain vasculature. Using the BTZ-based AIEgen **70**, 2P imaging of mouse brain tissues was achieved with high resolution and showed significant improvements, reaching a depth of 1064 μm at 1300 nm compared to 700 µm under 1PA (Table 3) (Qi et al., 2018). This highlights the sequential increased depth penetration of medical imaging using NIR-II wavelengths compared to shorter wavelengths, and the advantage of 2P over 1P microscopy. On a separate occasion, the same group used similar AIE NPs on a home built OPO laser-based system to demonstrate penetration depths of up to 750 μm at a shorter 2P wavelength (1040 nm) (Table 3) (Liu et al., 2021). Here, mouse brain vessels as small as 3.17 µm were imaged. The AIEdots prepared from compound **72** by Samanta *et al.* also allowed 800 μm brain tissue penetration under 2PE at 1040 nm (Table 3) (Samanta et al., 2021). Interestingly, record absorption capacity and unprecedented 2P brightness values (>10^4^ GM) were measured for the tetraphenylethene-BODIPY (**75**) AIEDots made by Wang *et al.*, but the reported penetration depth was not higher than in more recent studies (700 µm vs. 1200 µm) (Wang et al., 2015). As this is one of the older reports from these studies (2015) it is worth noting that newer technologies with optimum imaging systems could assist in taking this value to similar levels. The potential development of non-invasive imaging techniques using 2P NIR-II excitation was demonstrated by Wang *et al.* using polymer dots **64** as contrast agents (Wang et al., 2019b). The imaging of mice brains was achieved at an impressive penetration depth of 400 µm without craniotomy (intact skull) (Figure 8, a-l). The authors also showed the distinct resolution improvement using NIR-II light for 2P imaging when imaging at depths, when compared to light of shorter wavelengths (Figure 8, m). Among other classes of luminescent materials, *in vivo* 2P NIR-II imaging was shown for the first time using QDs as a contrast agent by Ni *et al.* with the PbS/CdS QDs **84** (Ni et al., 2022). Imaging depths of 220 µm were achieved in the mouse brain vasculature with a fibre laser operating at 1550 nm. Zhou *et al.* also demonstrated the potential of dual-functional dye **60** that combined 2PEF and ROS generation. 2P imaging of the probe was demonstrated using NIR-II irradiation and a white light laser was used to induce ROS generation in tumours of living mice, allowing for image-guided PDT (Zhou et al., 2021). A triphenylamine dipole was thus used as a theranostic agent to combine 2P imaging and cancer treatment in mice (Table 3).

Unsurprisingly, commercially available OPO laser-based microscopes are popular systems for imaging experiments; with most examples performed in the early NIR-II range (1000–1300 nm) (Table 3) although imaging at wavelengths above 1200 nm could show the optimum optical benefits. This highlights the limitation of a lack of commercial imaging systems in the NIR-IIb range.

## 4 Discussion and Perspectives

**FIGURE 9:**
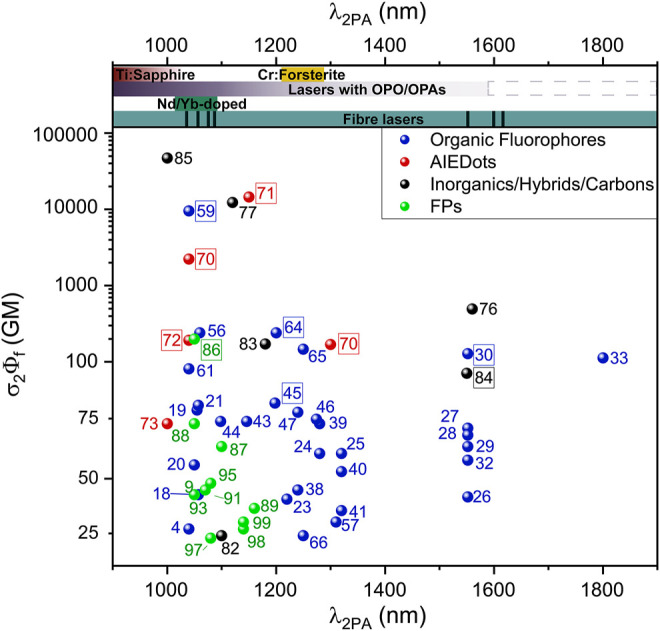
Plot of the 2P brightness and corresponding NIR-II wavelengths of the contrast agents presented in this review (σ_2_Φ_f_ > 24 GM). The 2P contrast agents used *in vivo* are represented with a framed number. For clarity, a linear scale for brightnesses between 0 and 100 GM, and a logarithmic scale above 100 GM. The typical range of wavelengths covered by common SWIR lasers is shown for reference (power attenuation is represented by the fading colour; note that OPOs and OPAs can extend past 2000 nm).

The design of OMI probes usable in the “biological transparency windows” is arguably one of the most prevalent challenges in optobiology today. Shifting the 1PE band of dyes to the NIR-II is possible, but highly challenging as massive bathochromic shifts often come at the cost of fluorescence quantum yield and brightness. To circumvent this problem, 2P NIR-II imaging shows outstanding potential for expanding the applications of fluorescence-based medical imaging. By reducing auto-fluorescence and scattering while confining the excitation to a femtoliter-sized volume, this technique allows for dramatic resolution improvement and enhanced deep tissue imaging which would help the applications of image-guided surgery, point-by-point chemical detection, and image-based diagnostics reach their full potential. A wide range of probe design strategies have been implemented recently to achieve sufficient 2P brightness (>25 GM) in this wavelength range (Figure 9). Organic fluorophores have the benefit of low toxicity and easy functionalisation to change their optical and biological properties. Among them, xanthene and polymethine dyes hold great potential for 2P NIR-II imaging due to their versatility allowing bathochromic shifts in their optical properties while retaining a highly emissive character. However, in designing such contrast agents, dye design rules and choice of fluorophore should be considered carefully as centrosymmetric molecules can easily fail to reach the NIR-II range because of selection rules. In contrast, significantly higher σ_2_Φ_f_ values are witnessed when 2P absorbers are integrated into nanoparticle structures, including AIE systems, achieving unprecedented 2P brightnesses of up to 5.6 × 10^5^ GM in the NIR-II. 2P NIR-II luminescent materials have the potential to expand into image guided surgery, diagnostics and chemical sensing due to the high resolution and penetration depths that can be reached, which can also expand to controlled photo-release in therapy applications (Sun et al., 2017a; Zhao et al., 2019). For *in vivo* imaging, unsurprisingly, AIEgens have proven to be the 2P dyes of choice. Such nanosystems also show good biocompatibility and high brightnesses and facilitate mouse brain vascular imaging at depths of up to 1200 µm, and even showed the potential for through-skull brain imaging. Nonetheless, such imaging agents require a certain amount of design and preparation, and more user-friendly solutions can be found in commercially available biological stains. Indeed, there is still to this day a significant gap in the availability and measurement of 2P properties of such dyes, even though many potential 2P dyes can be found in common cell stains (ICG, Alexa dyes etc.). Easily accessible and biologically compatible probes, and their custom-made derivatives, hold great potential in the development of 2P imaging in the NIR-II.

Such compounds, as well as synthetic fluorophores with higher NIR absorption capacities, have likely not yet been categorised due to the difficulty in taking such measurements in this optical window due to the lack of commercial 2P solutions. Nevertheless, more recently, commercially available OPO-based laser imaging systems have facilitated greater access to 2P NIR-II measurements and 2P imaging. These new systems have led to a dramatic rise in publications of *in vivo* studies emerging from 2015 and beyond, and will likely continue to be developed to push the field. Fibre lasers offer huge promise in this sense, by facilitating the necessary power requirements to encourage imaging at wavelengths above 1300 nm, and to give rise to a new generation of imaging agents and biological applications. In this highly favourable context, we now hope that this review will serve as a motivation for researchers to explore the vast possibilities of multiphoton-excited luminescence in the NIR-II transparency windows.
